# The effectiveness of newly synthesized quaternary ammonium salts differing in chain length and type of counterion against priority human pathogens

**DOI:** 10.1038/s41598-022-24760-y

**Published:** 2022-12-16

**Authors:** Natalia Kula, Łukasz Lamch, Bożena Futoma-Kołoch, Kazimiera A. Wilk, Ewa Obłąk

**Affiliations:** 1 Department of Physico-Chemistry of Microorganisms, Faculty of Biological Sciences, University of Wrocław, Przybyszewskiego 63/77, 51-148 Wrocław, Poland; 2 Department of Engineering and Technology of Chemical Processes, Faculty of Chemistry, Wrocław University of Science and Technology, Wybrzeże Wyspiańskiego 27, 50-370 Wrocław, Poland; 3 Department of Microbiology, Faculty of Biological Sciences, University of Wrocław, Przybyszewskiego 63/77, 51-148 Wrocław, Poland

**Keywords:** Antimicrobials, Pathogens

## Abstract

Quaternary ammonium salts (QAS) commonly occur as active substances in disinfectants. QAS have the important property of coating abiotic surfaces, which prevents adhesion of microorganisms, thus inhibiting biofilm formation. In this study, a group of nine monomeric QAS, differing in the structure and length of the aliphatic chain (C12, C14, C16) and the counterion (methylcarbonate, acetate, bromide), were investigated. The study included an analysis of their action against planktonic forms as well as bacterial biofilms. The compounds were tested for their anti-adhesion properties on stainless steel, polystyrene, silicone and glass surfaces. Moreover, mutagenicity analysis and evaluation of hemolytic properties were performed. It was found that compounds with 16-carbon hydrophobic chains were the most promising against both planktonic forms and biofilms. Tested surfactants (C12, C14, C16) showed anti-adhesion activity but it was dependent on the type of the surface and strain used. The tested compounds at MIC concentrations did not cause hemolysis of sheep blood cells. The type of counterion was not as significant for the activity of the compound as the length of the hydrophobic aliphatic chain.

## Introduction

Quaternary ammonium salts (QAS) are a broad group of cationic surfactants. Due to their unique structure and properties, they are widespread in most industries as well as being found in nature, where they can have protective functions during cell osmotic stress (e.g., glycine betaine produced by *Rhizobium meliloti*)^1,2^. They are used as surfactants in detergents, where they reduce the surface tension at phase interfaces. In addition, they can be components of laundry detergents, solvents, or anticorrosive preparations. They are also used during chemical synthesis as catalysts^1,2^. QAS are used in medicine as drugs, e.g., in anesthesiology, where they induce skeletal muscle relaxation (e.g., sugammadex, D-tubocurarine, toxiferine). There are also used to treat hyperhidrosis and salivation (e.g., glycopyrronium bromide)^3^.

QAS are particularly widely used as active substances in disinfectants^4–6^. Depending on their structure, they have antimicrobial activity against Gram-positive and Gram-negative bacteria, fungi and enveloped viruses. These include benzalkonium chlorides (BAC) and Decon7, a new generation of compounds that eradicate biofilms of *Staphylococcus* spp. and *Pseudomonas* spp.^7^. Research in recent years has shown that salts included in disinfectants effectively inactivate the SARS-CoV-2 virus^7^. The precise mechanism of action of QAS is not fully understood. It is generally thought that the compounds can interact with the cell membranes of microorganisms, inducing their disintegration. Depending on their chemistry, QAS can interact with protein or genetic material^8,9^.

QAS has an amphiphilic structure due to the presence of a hydrophilic (lipophobic) head and a hydrophobic (lipophilic) tail that shows affinity to cell membrane lipids. These surfactants also have counterions (e.g. bromide—Br^−^, chloride—Cl^−^), which were shown to increase the antimicrobial activity of the compound^10–12^.

The traditional QAS structure bears a positively charged hydrophilic (lipophobic) moiety, hydrophobic (lipophilic) tail, mostly comprising a shorter or longer alkyl fragment, and negatively charged counterions. One of the most challenging drawbacks connected with widespread use of some types of cationic surfactants is their limited biodegradability connected with toxicity to aquatic organisms^13^. Easily biodegradable and less toxic surfactants very often include an appropriate labile moiety in their structures: commonly ester or amide^14^. Chemically and/or enzymatically induced cleavage of the labile bond will cause the separation of the polar part and the hydrophobic tail and, as a consequence, change of surface activity^15^. Typically, counterions for QAS are halides (especially chlorine and bromide) or methylsulfate anions. On the other hand, there is a great demand for novel types of counterions meeting the requirements of safe disinfecting agents and the principles of “green chemistry”, especially lactates, formates and acetates. QAS derivatives, containing the aforementioned novel counterions, are synthesized by quaternization of an appropriate tertiary amine derivative with dimethyl carbonate, followed by reaction with acid (e.g. acetic, formic or lactic) stronger than carbonic. The semi-products—amphiphilic quaternary methylcarbonates—may be isolated and purified, since they comprise a valuable group of reactive surfactants, prone to counterion exchange, especially in biological systems^16,17^.

Biofilm formation by microorganisms is a common problem in hospital and industrial environments. Biofilm is estimated to cause about 80% of severe human infections, with a high mortality rate^10^. Bacteria and fungi, both pathogenic and non-pathogenic species, have the ability to form consortia. Microorganisms of great epidemiological significance are MDR (multidrug-resistance) microorganisms, which include *Pseudomonas aeruginosa* and methicillin-resistant *Staphylococcus aureus* (MRSA)^10,18,19^. Implant-related infections are a special threat in the hospital environment. They are estimated to account for 60–70% of all hospital-acquired infections. Pathogenic microorganisms show the ability to form biofilms on materials that are implanted in the body—plastic, stainless steel, silicone. The materials are used to make endotracheal tubes, heart valves, knee endoprostheses, and implants^19^. Biofilms formed by microorganisms in the endotracheal tube are a common cause of ventilator-associated pneumonia, which is one of the most common infections in intensive care units and the leading cause of death for intubated patients^20^.

The first step in biofilm formation by microorganisms is cell adhesion to surfaces. Studies show that QAS can coat plastics, therefore preventing microbial adhesion to surfaces. It has been documented that QAS are also capable of destroying biofilms that have already been formed^21–23^.

The widespread use of QAS in many fields has resulted in the occurrence of resistance of microorganisms to these compounds, and hence the need to synthesize new chemical structures of disinfectants^24–26^. A group of novel QAS type surfactants, comprising a tertiary amide linker and bromide, methylcarbonate and acetate counterion, have been designed, synthesized and carefully analyzed. These custom designed products possess optimal structural features—a tertiary amide linking group for moderation of hydrogen bonding within the surfactant molecule enabling improved aqueous solubility, different counterions, including soft and reactive ones, balanced chemical stability and biodegradability, as well as synthetic routes meeting at least some of the principles of “green chemistry”—for application as disinfectants. In order to carefully study the biological performance of the engineered surfactants we synthesized compounds containing different alkyl chain lengths (*n*-dodecyl, *n*-tetradecyl and *n*-hexadecyl) and selected counterions of different characteristics: acetate (CH_3_COO^−^), containing a less hydrating moiety in comparison to bromide (Br^−^), and membrane reactive methylcarbonates (CH_3_CO_3_^−^). A group of monomeric QAS with different lengths of aliphatic chains and counterions was synthesized in order to select the most effective disinfectants. We hypothesize that chain length and type of counterion can affect the antimicrobial activity. QAS activity against planktonic forms and biofilms of bacteria and fungi was studied. The compounds were also tested for their rate of action against the biofilm formed. It was verified whether the tested QAS exhibit an antiadhesive effect, preventing biofilm formation. These compounds could be used in medicine as effective disinfectants or fungicides. In addition, the mutagenic and hemolytic properties of QAS were evaluated.

## Results

### Design, synthesis, identification, and characteristics of novel QAS surfactants

**Figure 1:**
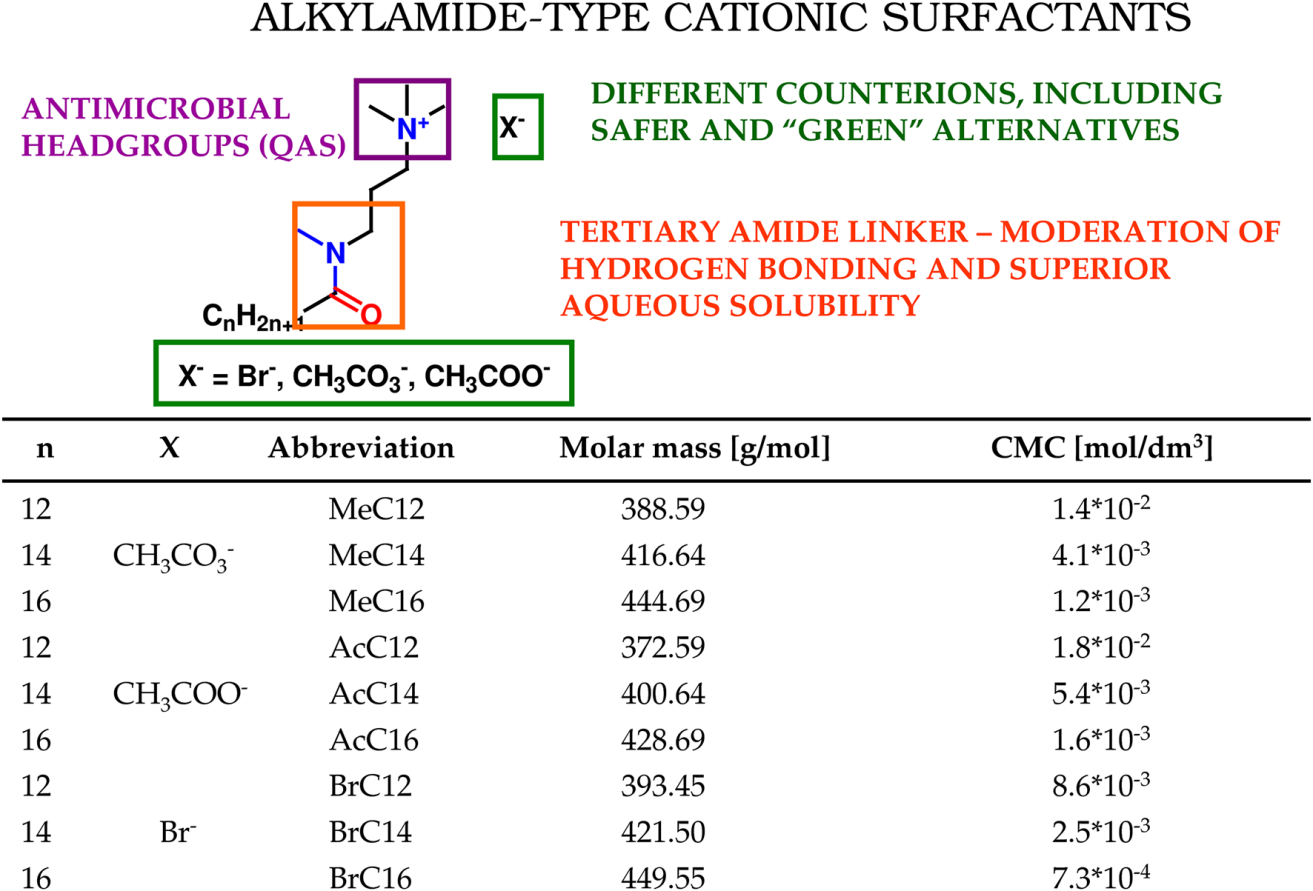
Structures, abbreviation, and properties of the studied surfactants.

The design of novel alkylamide-type QAS surfactants took into account three main aspects: moderate biodegradability assuming both safety and sufficient stability during storage/usage; appropriate aqueous solubility, especially for the most effective long alkyl chain derivatives, as well as different counterion types, including “green” and reactive ones. Therefore, our rational products comprised an antimicrobial QAS headgroup, a tertiary amide linker without N–H bonds for moderation of hydrogen bonding, superior aqueous solubility, and balanced chemical stability/biodegradability as well as three different counterions: bromide, acetate and methylcarbonate. The design and some basic properties of the studied surfactants are shown in Fig. 1.

According to cationic surfactants described in the literature, we designed and synthesized novel antimicrobial cationic surfactants of linear (single-head, single-tail) structure with an appropriate cleavable tertiary amide linker as well as counterions meeting the requirements of safe disinfecting agents and the principles of “green chemistry”. Our surfactants meet all above-mentioned aspects of novel multifunctional amphiphiles: synthetic requirements of sustainable chemistry with potential for technological applications, avoidance of toxic intermediates and excellent water solubility. Taking into consideration possible quaternizing agents, e.g. alkyl halide, dimethyl sulfate or dimethyl carbonate, we chose methyl bromide—a standard intermediate for QAS synthesis, enabling very mild synthetic conditions—and a very potent “green chemistry alternative”: dimethyl carbonate. Moreover, monomethyl carbonates—yielded in reaction of appropriate hydrophobic intermediate with dimethyl carbonate—may constitute raw materials for surfactants with novel, biocompatible counterions, as acetate and lactate groups^16,17^. Generally, an excess of quaternizing agent is needed to achieve a sufficient reaction rate and appropriate yield of the desired product. That is why one of the main advantages of dimethyl carbonate as a quaternizing agent is its optimal boiling point (around 90 °C) enabling easy removal after reaction by evaporation under reduced pressure, followed by reuse for synthesis as well as reduced risk of accidental evaporation due to reaction heat^27^. In contrast to dimethyl sulfate, a toxic, mutagenic, and carcinogenic quaternizing agent with moderate stability in the environment, dimethyl carbonate does not constitute a risk connected with its presence as an impurity in the given product.

The structures of the obtained QAS-type surfactants were confirmed by ^1^H NMR measurements, while values of critical micelle concentrations were assessed by conductrometric measurements (see detailed description in Electronic Supplementary Material, Figs. S10–S12). In general, all ^1^H NMR spectra of the studied surfactants (see spectra in Electronic Supplementary Material, Figs. S13–S15 and Tables 3, 4, 5 for detailed information) showed characteristic signals, attributed to methyl groups at the end of the alkyl chain and within the tertiary amide linker (around 0.85–0.9 ppm and 3.05–3.1 ppm, respectively). Moreover, a strong singlet of three methyl moieties within the tertiary ammonium headgroup was visible at 3.3–3.4 ppm. For methylcarbonates and acetates, signals, overlapping with methylene groups neighboring nitrogen atoms within linkers, were visible at 3.45–3.65 ppm. Values of critical micelle concentrations for three groups of surfactants, i.e. methylcarbonates, bromides and acetates, followed the Stauff–Klevens rule and were within the range 10^–2^–10^–4^ M for C12–C16 derivatives, corresponding to typical values of linear-type ionic surfactants^28^.

### Minimal inhibitory concentration (MIC) and minimal bactericidal concentration (MBC)

Minimal inhibitory concentration (MIC) and minimal bactericidal concentration (MBC) were determined to evaluate the activity of the newly synthesized QAS. The tested compounds differed in the structure of the counterion (Me—methylcarbonate, Ac—acetate, Br—bromide) and the length of the aliphatic chain (C12, C14, C16). The activity of the compounds was analyzed against Gram-positive strains: *S. epidermidis* ATCC 35984, *S. epidermidis* B374, *S. epidermidis* SI8, *S. aureus* ATCC 6538, *S. aureus* MRSA R98 and Gram-negative: *P. aeruginosa* ATCC 27853, *P. aeruginosa* PAO1, *E. coli* ATCC 11229 and *E. coli* H64.

The structure of the surfactant was shown to affect its antimicrobial activity—the compounds with 14 and 16 carbon atoms in the aliphatic chain were more effective against Gram-positive bacteria that those with 12 carbon atoms. Among Gram-negative bacteria *P. aeruginosa* ATCC 27,853 and clinical strain were less susceptible to tested chemicals. As regards type of counterion, the most effective were methylcarbonate and bromide with C16 hydrophobic chains. Tested compounds were less effective against Gram-negative bacteria. The lowest MIC was obtained for *E. coli* H64, which for the MeC14 and MeC16 compounds was 80 µM. Against *E. coli* ATCC 11,229, acetate with 16 carbon atoms in the chain showed the greatest effectiveness. It caused growth inhibition of the strain at 80 µM. None of the QAS tested showed effectiveness for *P. aeruginosa* strains from the ATCC collection and the clinical strain. Among Gram-positive bacteria the most sensitive were *S. epidermidis* B374, *S. epidermidis* SI8, *S. aureus* ATCC 6538, *S. aureus* MRSA R98 against which the compounds with 16 carbon atoms in the chain showed activity in concentrations of 10 µM (p < 0.004) (Table 1).

**Table 1:** Minimal inhibitory concentration (MIC) and minimal bactericidal inhibitory concentration (*MBC) monomeric quaternary ammonium salts (QAS) with different counterion (Me—methylcarbonates, Ac—acetates, Br—bromides) and aliphatic chain length (C12, C14, C16).

Strain	Compound								
	Minimal inhibitory concentration (MIC) and minimal bactericidal concentration (*MBC) of tested compounds [µM]								
	MeC12	MeC14	MeC16	AcC12	AcC14	AcC16	BrC12	BrC14	BrC16
*S. epidermidis* ATCC 35984	320 *640	40 *40	40 *40	640 *640	160 *160	640 *640	640 *640	160 *160	640 *640
*S. epidermidis* B374	640 *160	80 *160	80 *160	320 *320	40 *40	40 *40	640 *640	640 * > 640	10 *10
*S. epidermidis* SI8	320 *640	40 *40	10 *10	640 *640	160 *160	10 *10	640 *640	160 *160	10 *10
*S. aureus* ATCC 6538	320 *640	80 *80	10 *10	1280 *1280	160 *160	80 *80	1280 *1280	320 *320	320 *320
*S. aureus* MRSA R98	640 *640	80 *80	40 *40	640 *640	160 *160	10 *10	640 *640	320 *320	320 *320
*E. coli* ATCC 11229	640 *640	320 *320	320 *320	1280 *1280	320 *320	80 *160	1280 *1280	> 1280 * > 1280	640 *1280
*E. coli* H64	640 *640	80 *80	80 *80	1280 *1280	160 *160	160 *320	1280 *1280	320 *320	160 *160
*P. aeruginosa* ATCC 27853	> 1280 * > 1280	1280 * > 1280	> 1280 * > 1280	> 1280 * > 1280	1280 * > 1280	> 1280 * > 1280	1280 *1280	1280 *1280	> 1280 * > 1280
*P. aeruginosa* PAO1	> 1280 * > 1280	> 1280 * > 1280	> 1280 * > 1280	> 1280 * > 1280	> 1280 * > 1280	> 1280 * > 1280	> 1280 * > 1280	> 1280 * > 1280	> 1280 * > 1280

### Adhesion

**Figure 2:**
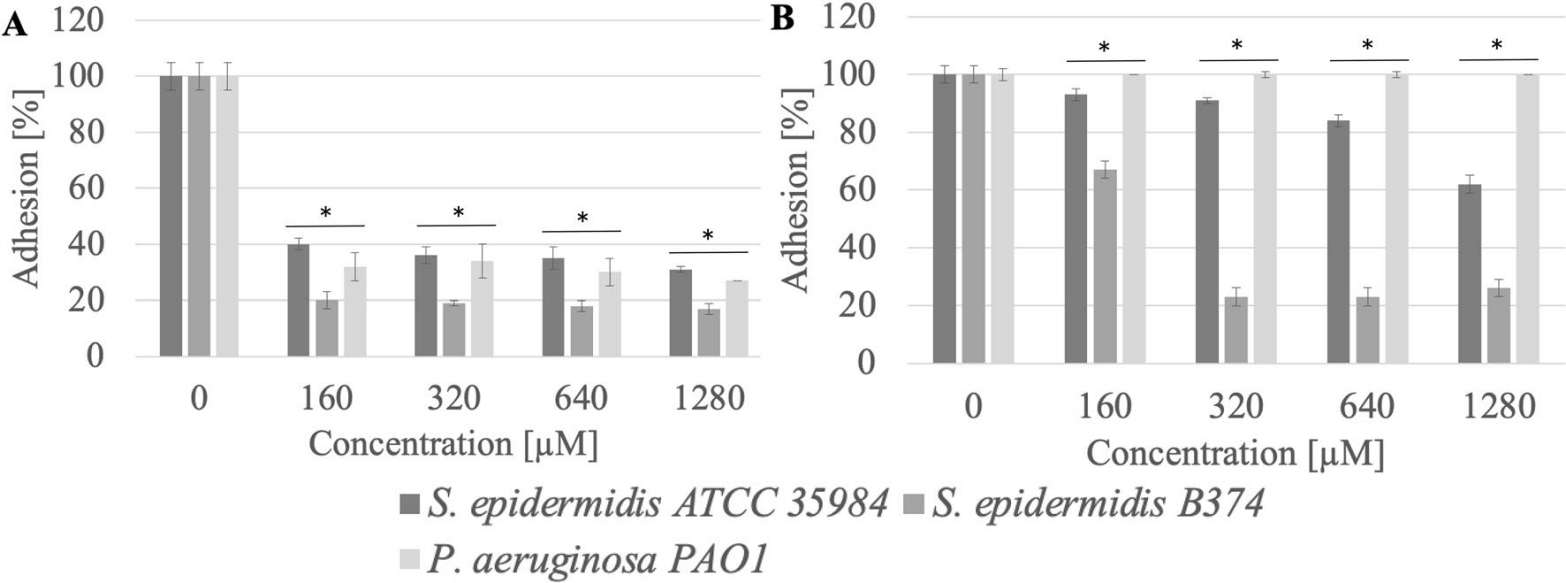
Adhesion of microorganisms to surfaces ((**A**) stainless steel, (**B**) polystyrene) after incubation with MeC16 compound; *significant difference between groups (p < 0.05).

**Figure 3:**
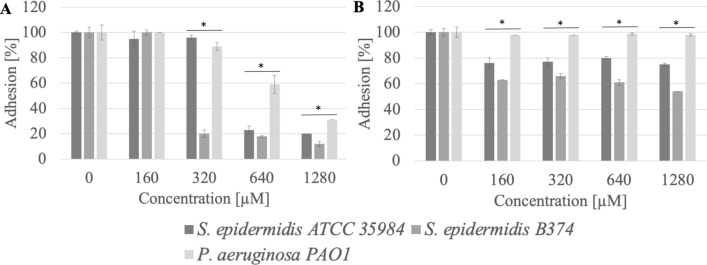
Adhesion of microorganisms to surfaces ((**A**) stainless steel, (**B**) polystyrene) after incubation with MeC12 compound; *significant difference between groups (p < 0.05).

**Figure 4:**
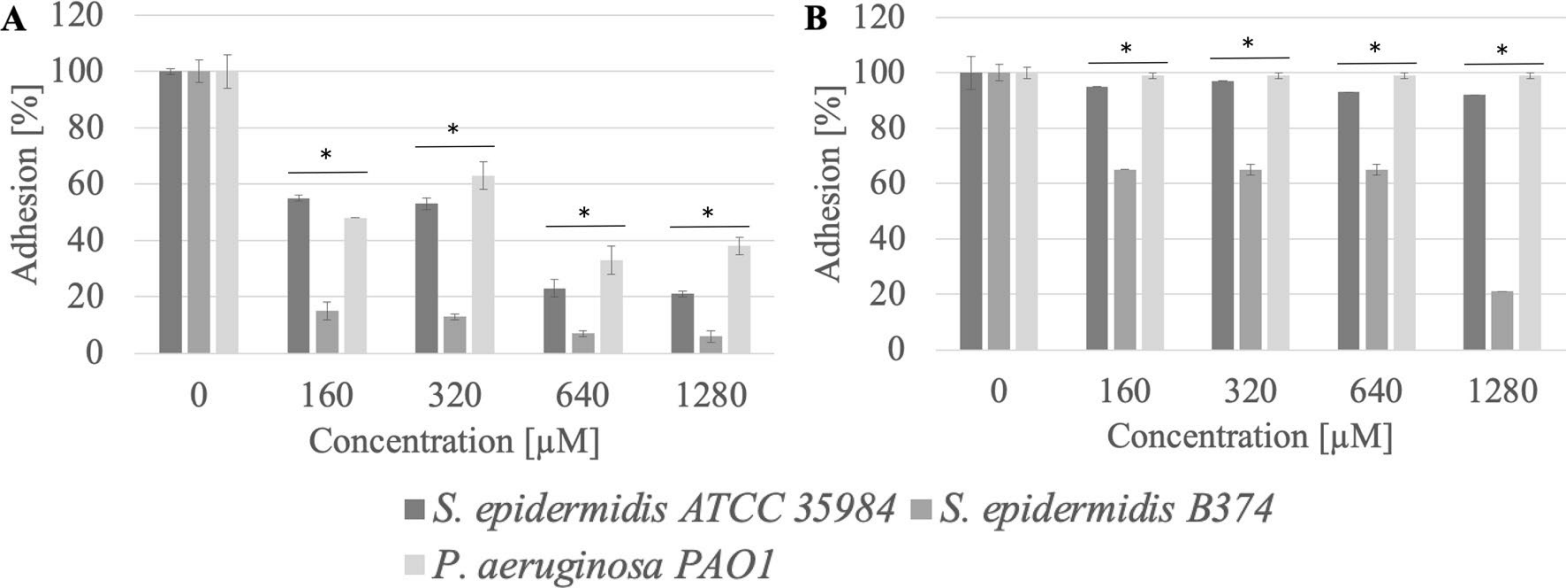
Adhesion of microorganisms to surfaces ((**A**) stainless steel, (**B**) polystyrene) after incubation with MeC14 compound; *significant difference between groups (p < 0.05).

**Figure 5:**
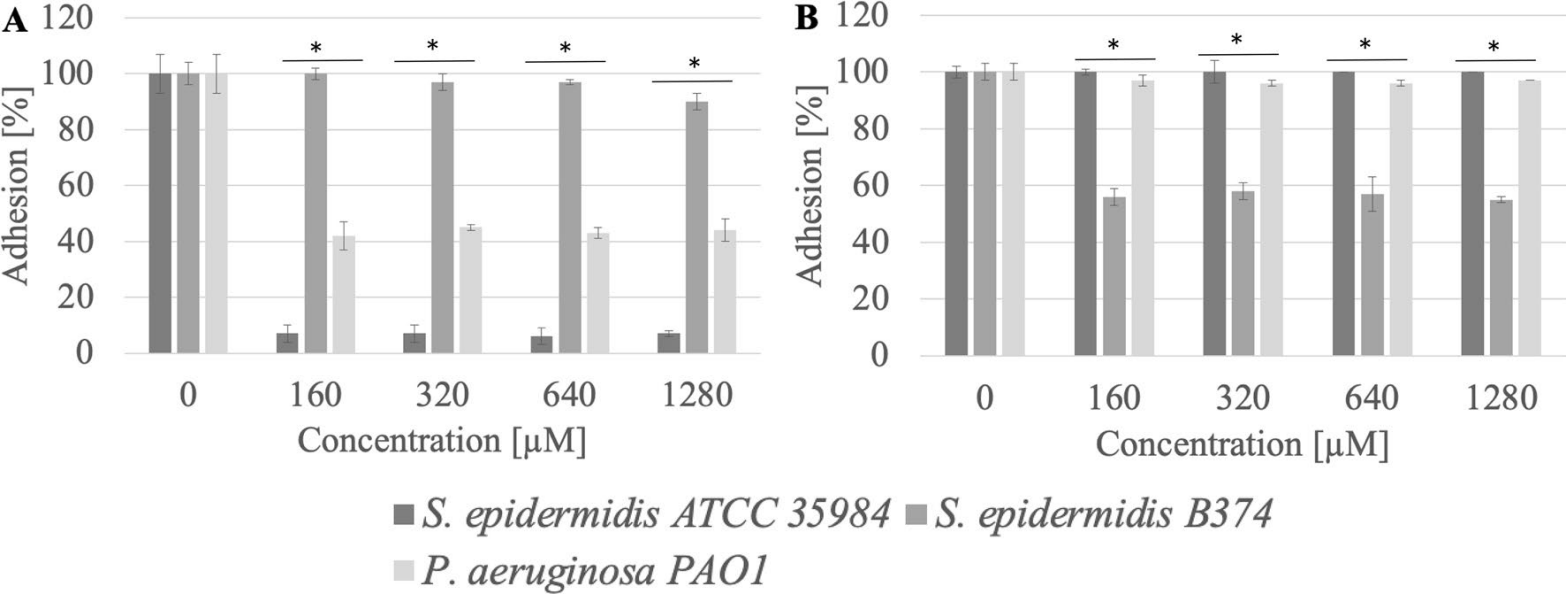
Adhesion of microorganisms to surfaces ((**A**) stainless steel, (**B**) polystyrene) after incubation with AcC16 compound; *significant difference between groups (p < 0.05).

**Figure 6:**
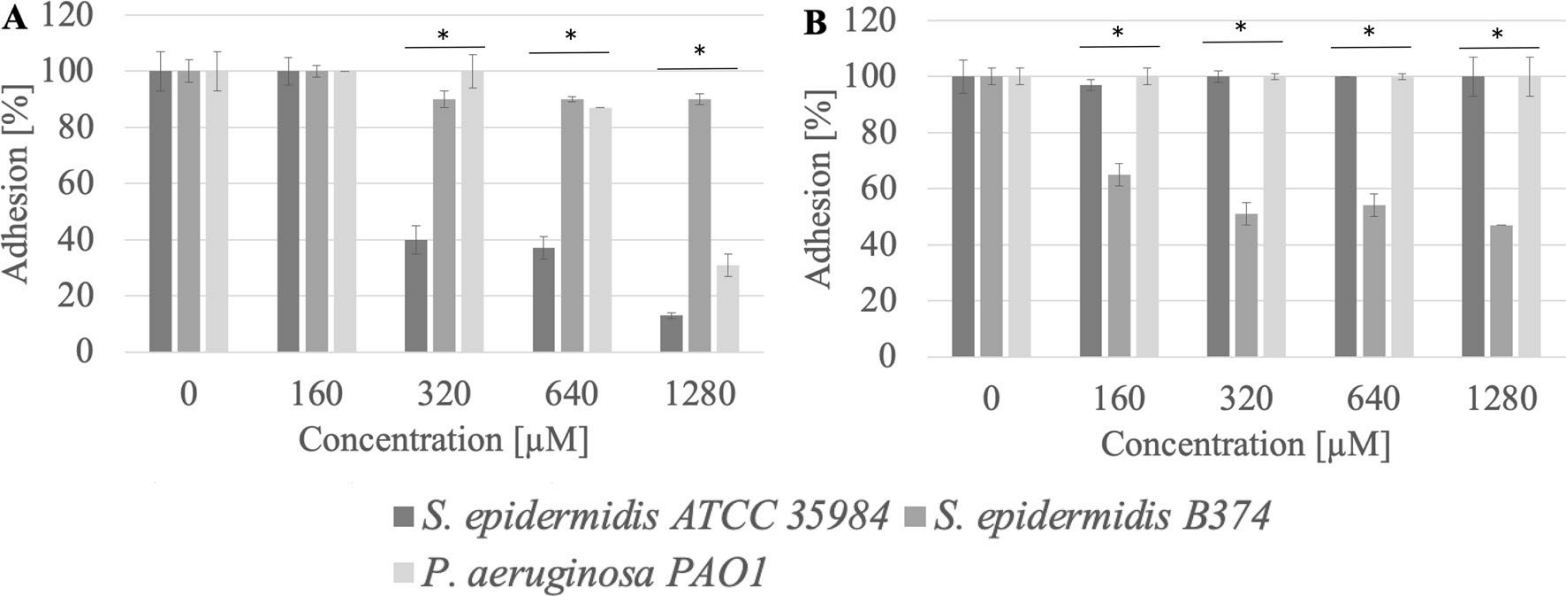
Adhesion of microorganisms to surfaces ((**A**) stainless steel, (**B**) polystyrene) after incubation with AcC12 compound; *significant difference between groups (p < 0.05).

**Figure 7:**
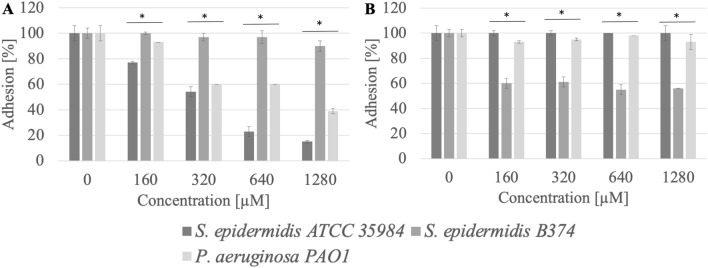
Adhesion of microorganisms to surfaces ((**A**) stainless steel, (**B**) polystyrene) after incubation with AcC14 compound; *significant difference between groups (p < 0.05).

**Figure 8:**
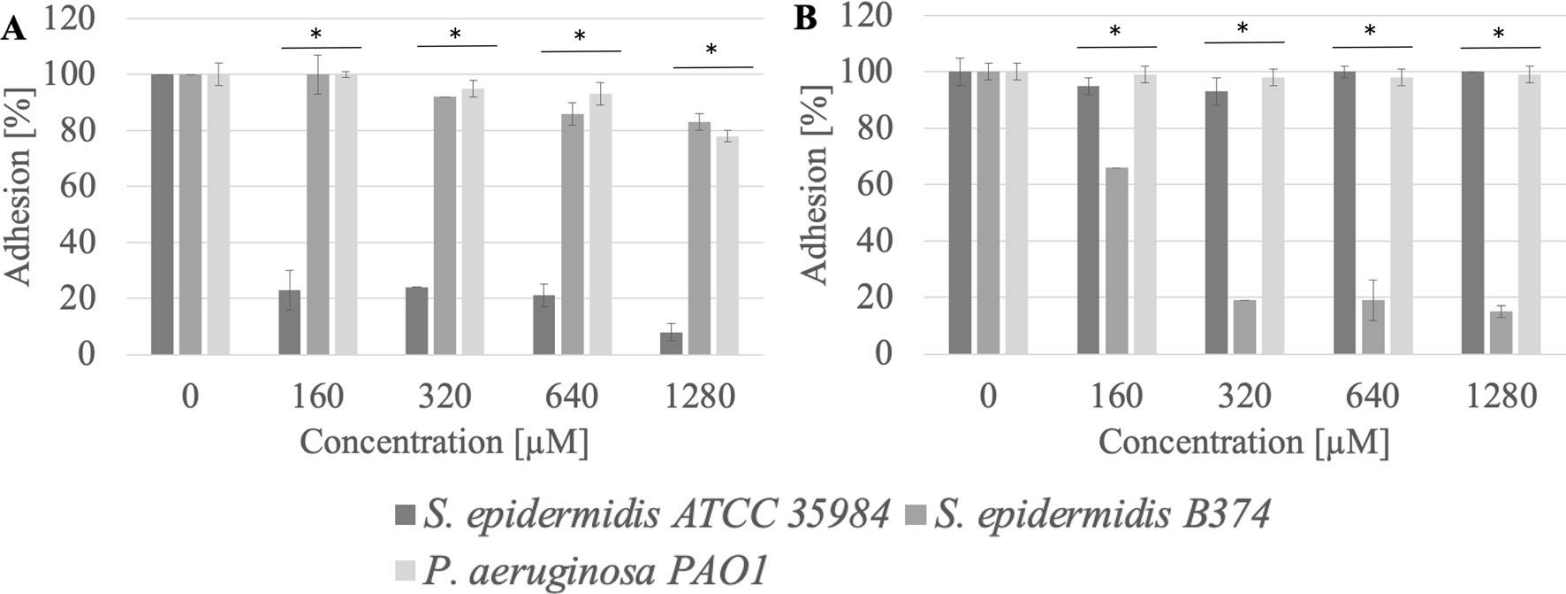
Adhesion of microorganisms to surfaces ((**A**) stainless steel, (**B**) polystyrene) after incubation with BrC16 compound; *significant difference between groups (p < 0.05).

**Figure 9:**
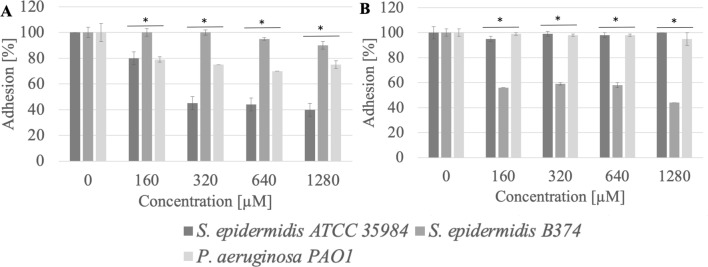
Adhesion of microorganisms to surfaces ((**A**) stainless steel, (**B**) polystyrene) after incubation with BrC12 compound; *significant difference between groups (p < 0.05).

**Figure 10:**
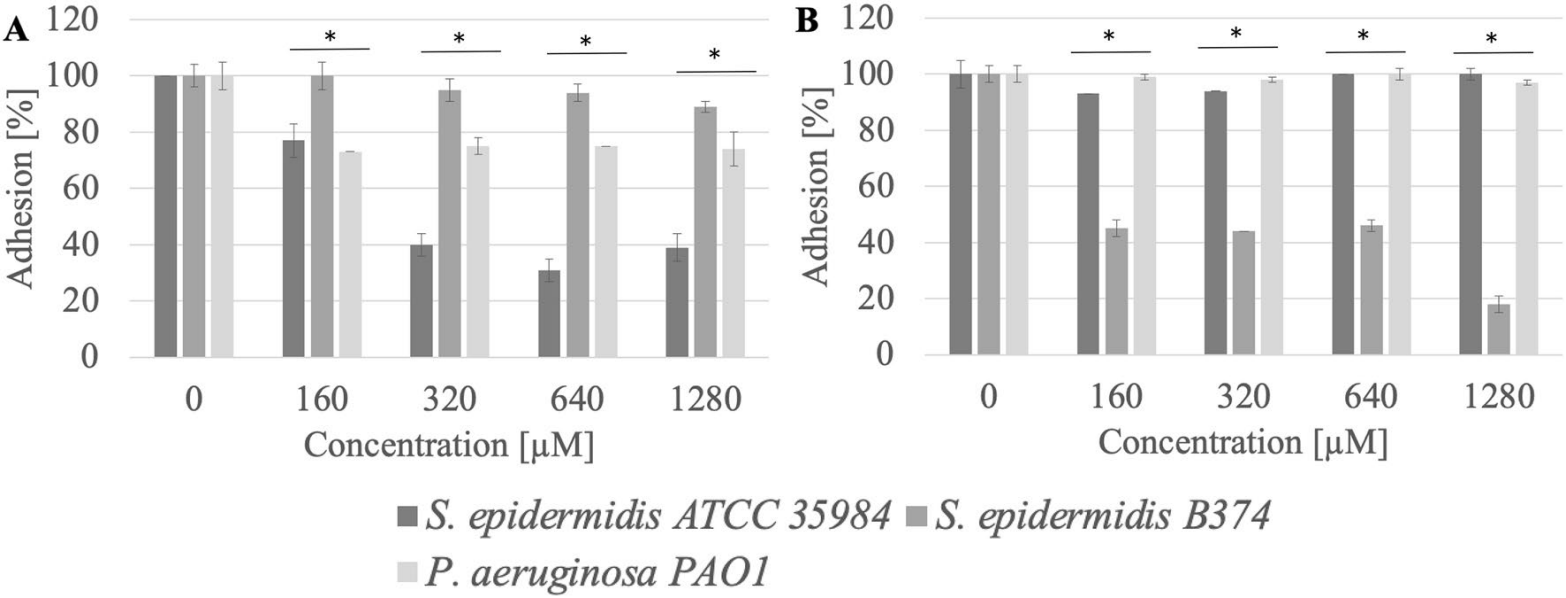
Adhesion of microorganisms to surfaces ((**A**) stainless steel, (**B**) polystyrene) after incubation with BrC14 compound; *significant difference between groups (p < 0.05).

Adhesion of microbial cells is the first step in biofilm formation. QAS, due to their amphiphilic structure, may be used to coat various surfaces, thus preventing microbial cell adhesion and biofilm formation. The study proved that compounds with 16 carbon atoms in the chain were more effective in inhibiting the adhesion of pathogenic strains to the tested surfaces (polystyrene, stainless steel) in comparison to QAS with 12 or 14 carbon atoms in the chain (Figs. 2, 3, 4, 5, 6, 7, 8, 9, 10). There was no correlation between the structure of the counterion and the anti-adhesion effect.

It was observed that methylcarbonates with a 16-carbon aliphatic chain (MeC16) inhibited the adhesion to the stainless steel surface of *S. epidermidis* ATCC 35984, *S. epidermidis* B374, and *P. aeruginosa* PAO1 at a level of almost 69–83% (p < 0.004). Reduced adhesion to the polystyrene surface after treatment with MeC16 at a concentration of 320 µM (0.03 CMC) was exhibited by *S. epidermidis* B374. The reduction in adhesion was estimated at the level of 77% (p < 0.004) (Fig. 2).

It was observed that methylcarbonates with a 12-carbon aliphatic chain (MeC12) at a concentration of 1280 µM (0.09 CMC) inhibited the adhesion to the stainless steel surface of *S. epidermidis* ATCC 35984, *S. epidermidis* B374, and *P. aeruginosa* PAO1 at a level of 69–88% (p < 0.004). Reduced adhesion to the polystyrene surface after treatment with this chemical (1280 µM) for *S. epidermidis* ATCC 35984 and *S. epidermidis* B374 was 25–46% (p < 0.003) (Fig. 3).

The anti-adhesion effect was also demonstrated by methylcarbonate with a 14-carbon aliphatic chain (MeC14). This surfactant significantly reduced the adhesion of three tested strains to the surface of stainless steel and in a less extent on polystyrene (Fig. 4). Adhesion reduction at a concentration of 1280 µM (0.3 CMC) was 62–94%.

The 16-carbon hydrophobic chain acetate (AcC16) also showed high anti-adhesion efficiency, reducing the adhesion of *S. epidermidis* ATCC 35984 to the stainless steel surface at a concentration of 160 µM (0.1 CMC) by approximately 93% (p < 0.004), while the adhesion of *P. aeruginosa* PAO1 was reduced by 58% (p = 0.04) (Fig. 5). Application of AcC16 compound at a concentration of 160 µM (0.1 CMC) reduced the adhesion to the polystyrene surface of *S. epidermidis* strain B374 by approximately 44% (p = 0.01) (Fig. 5).

Compounds that effectively reduce adhesion to stainless steel surfaces can also include acetate with a 12-carbon hydrophobic chain (AcC12). After its application in 1280 µM (0.07 CMC), the adhesion of strains: *S. epidermidis* ATCC 35984 and *P. aeruginosa* PAO1 was from 13 to 30% (p = 0.04) (Fig. 6).

A compound with 14-carbon atoms in the hydrophobic chain (AcC14, 1280 µM, 0.2 CMC) had similar activity against *P. aeruginosa* PAO1 cultivated on stainless steel than AcC12. After incubation with this compound, 1280 µM (0.2 CMC)) the adhesion of *S. epidermidis* ATCC 35984 was 15% (p = 0.01) (Fig. 7).

Quaternary ammonium salt BrC16 at a concentration of 160 µM (0.2 CMC) reduced the adhesion of *S. epidermidis* ATCC 35984 (p = 0.01) to a stainless steel surface by 77%. The inhibition of *S. epidermidis* B374 and *P. aeruginosa* PAO1 adhesion was 17–22% (p < 0.03) (1280 µM (1.8 CMC)) (Fig. 8).

Furthermore, it was found that the application of the compound (BrC16) inhibited the adhesion of *S. epidermidis* B374 to the polystyrene surface by approximately 85% at a concentration of 160 µM (0.2 CMC) (p = 0.04). This compound did not reduce the adhesion of the other strains to this surface (Fig. 8).

12 and 14-carbon bromide compounds also showed efficacy 1280 µM against *S. epidermidis* ATCC 35984, where they reduced adhesion to stainless steel at levels of 60% (p = 0.04) (Figs. 9, 10). *S. epidermidis* B374 strain had the weakest adhesion on the surface of polystyrene—42% (p < 0.001) (Fig. 9). QAS BrC14 was most effective in reducing adhesion to polystyrene for *S. epidermidis* B374 at a concentration of 1280 µM (0.5 CMC). It was reduced at the level of 82% (p = 0.02) (Fig. 10).

Adhesion to silicone and glass is described in Electronic Supplementary Material (Figs. S1–S9). Adhesion to these surfaces, in most cases, stayed high, regardless of the used QAS. Tested compounds inhibited adhesion to silicone at the level of 20–40% (p < 0.004). MeC16, MeC12, BrC16, BrC14, and BrC12 reduced adhesion *P. aeruginosa* PAO1 to glass by approximately 20% (p < 0.004).

### Biofilm eradication

QAS are capable of destroying mature bacterial and fungal biofilms. These compounds penetrate the formed biofilm and destroy biofilm structure.

**Figure 11:**
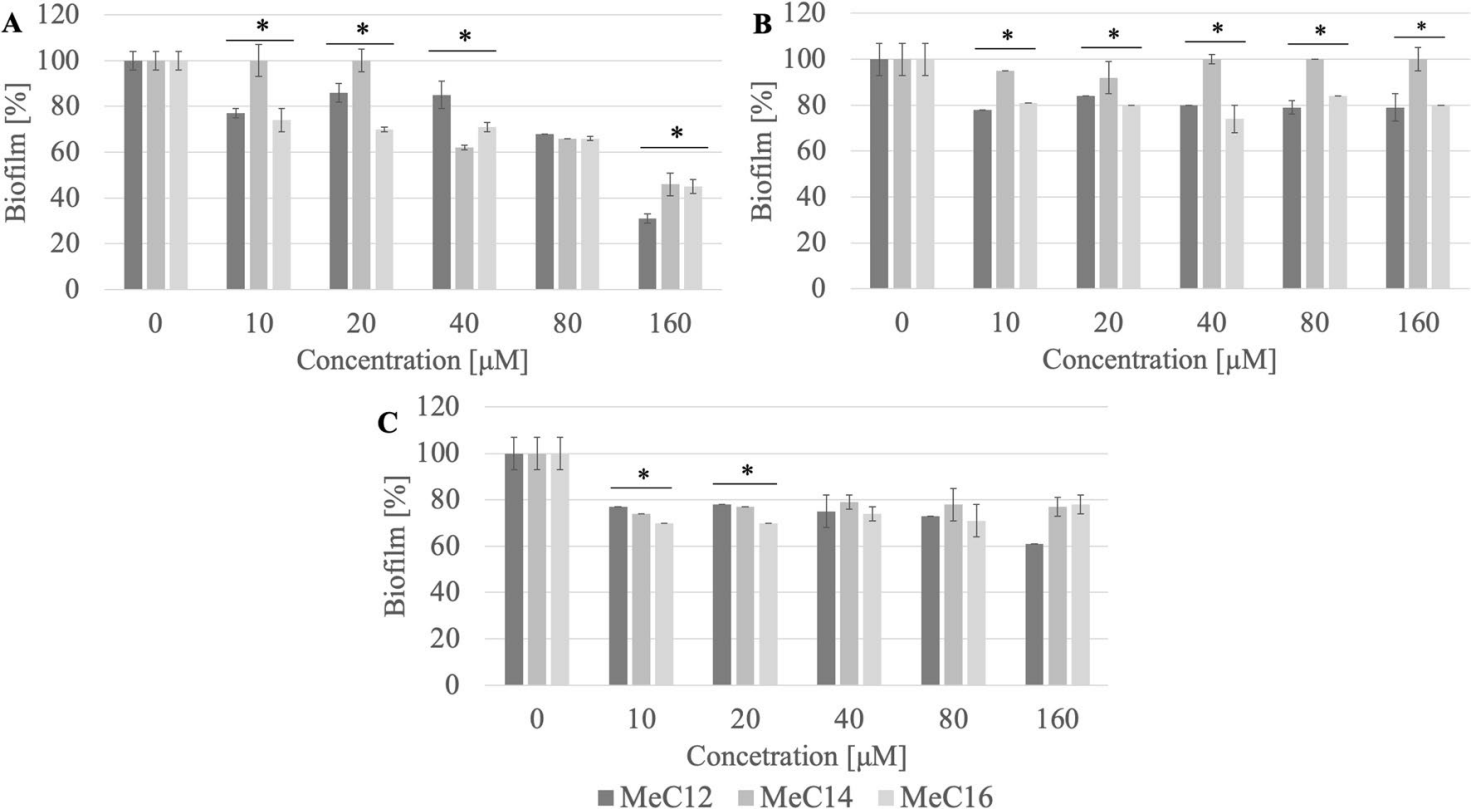
Biofilm eradication ((**A**) *S. epidermidis* B374, (**B**) *P. aeruginosa* PAO1, (**C**) *S. epidermidis* ATCC 35984) after incubation with selected QAS; *significant difference between groups (p < 0.05).

**Figure 12:**
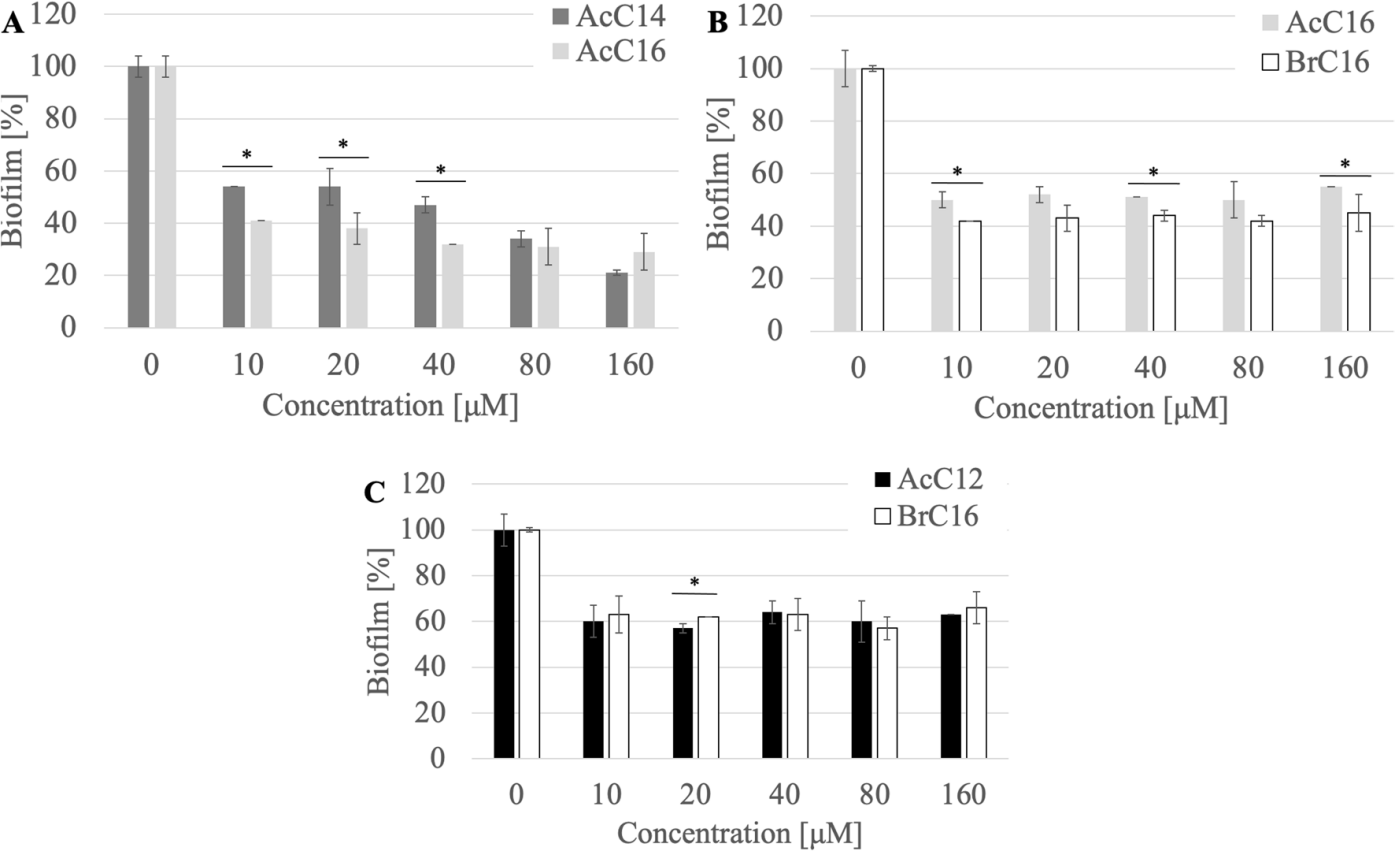
Biofilm eradication ((**A**) *S. epidermidis* B374, (**B**) *P. aeruginosa* PAO1, (**C**) *S. epidermidis* ATCC 35984) after incubation with selected QAS; *significant difference between groups (p < 0.05).

No correlation was observed between the structure of the counterion and the biofilm eradication activity. However, compounds with a methylcarbonate counterion showed less activity (Fig. 11). The study showed that compounds with 14 and 16 carbon atoms in the chain are more active. With the exception of biofilm formed by *S. epidermidis* ATCC 35,984 against which QAS with 12 carbon atoms in the chain was effective. The surfactants AcC14 and AcC16 eradicated the biofilm of *S. epidermidis* B374 by 70–80% at a concentration of 160 µM (0.03 CMC; 0.1 CMC respectively) (Fig. 12). Against *P. aeruginosa* PAO1 biofilm, the most effective compounds were AcC16 and BrC16, which at a concentration of 20 µM (0.01 CMC; 0.03 CMC respectively) showed 50–60% eradication of the biofilm. The tested surfactants AcC12 and BrC16 caused approximately 40% biofilm destruction of *S. epidermidis* ATCC 35984 at a concentration of 10 µM (0.0006 CMC; 0.01 CMC respectively).

### Biofilm-oriented antiseptics test (BOAT)

**Figure 13:**
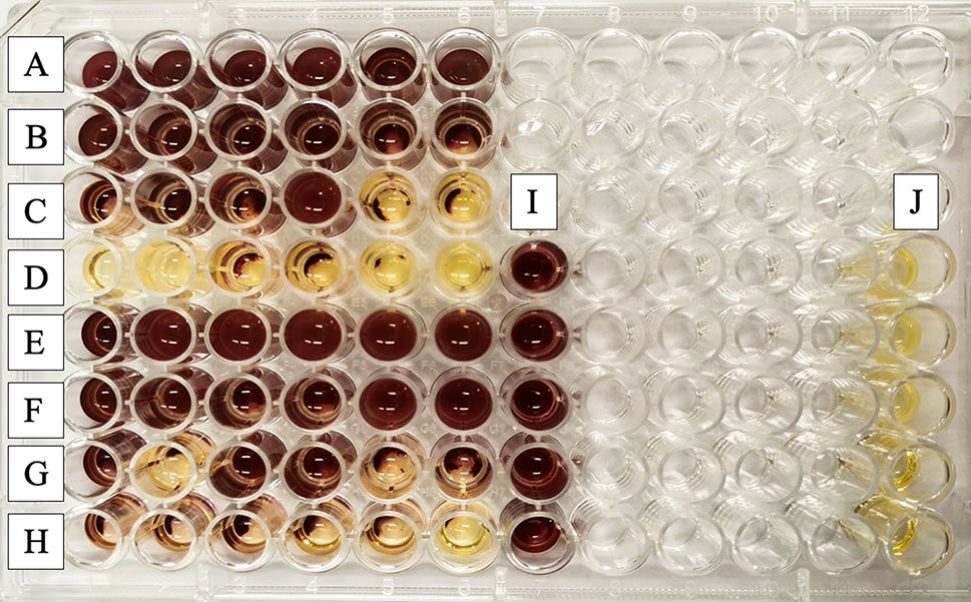
Biofilm Targeted Antiseptic Test (BOAT). The bottoms of the wells on the polystyrene microplate are covered with bacterial biofilm. A dark red coloration of the wells indicates that the bacterial biofilm survived treatment with the compound. The absence of red coloration is an indication of eradication of the biofilm formed in the well. (**I**) positive control (untreated biofilm), confirming the ability of the test strain to form biofilm; (**J**) negative control wells filled with medium (no biofilm) confirming the sterility of the test performed; (**A**–**D**) biofilm treated with the compound at the specified concentration (640, 320, 160, 80 µM respectively) for 60 min; (**E**–**H**) biofilm treated with the compound at the specified concentration (640, 320, 160, 80 µM respectively) for 30 min.

Among disinfectants, compounds are not only effective against microorganisms in low concentrations but are also characterized by fast action. For this purpose, the biofilm-oriented antiseptics test (BOAT) was performed. It was found that the analyzed compound (AcC16) showed efficacy against *S. epidermidis* B374 biofilm after 30 min of incubation (640 µM; 0.2 CMC), causing a decrease in cell survival of up to 30% and up to 9.5% after 60 min (Fig. 13). AcC16 was chosen for its most effective activity against *S. epidermidis* B374, *P. aeruginosa* PAO1.

### Mutagenicity

To evaluate the potential future application of the compounds, an analysis of their mutagenic properties is necessary. It was observed that among the nine QAS tested with different counterions (Me, Ac, Br) and chain lengths (C12, C14, C16), only BrC12 and BrC16 caused a reading frame shift type mutation. Their mutagenicity index against the strain *S*. Typhimurium TA98 was above 1.7 (MR > 1.7). This indicates the potential mutagenic properties of the QAS analyzed. These compounds did not cause base pair substitution type mutations, as their mutagenicity index against *S.* Typhimurium TA100 did not exceed the value of 1.7 (MR < 1.7). Other compounds tested do not have mutagenic potential (Table 2).

**Table 2:** Mutagenic properties of QAS with different counterion (Me, Ac, Br) and chain length (C12, C14, C16); ± SD.

*Salmonella* typhimurium TA98				*Salmonella* typhimurium TA100		
Compound	Concentration	Number of colonies	Mutagenicity index	Concentration	Number of colonies	Mutagenicity index
MeC12	½ MIC	21 ± 3.6	0.92	½ MIC	264 ± 11.3	0.94
	¼ MIC	17 ± 3.6	0.75	¼ MIC	208 ± 17	0.74
MeC14	½ MIC	21.7 ± 3.5	0.95	½ MIC	310 ± 17.3	1.10
	¼ MIC	13 ± 2.8	0.57	¼ MIC	329.5 ± 20.5	1.17
MeC16	½ MIC	4.3 ± 3.1	0.19	½ MIC	306.7 ± 11.5	1.09
	¼ MIC	9 ± 2	0.39	¼ MIC	305 ± 8.7	1.09
AcC12	½ MIC	20.8 ± 4.4	0.91	½ MIC	304 ± 11.3	1.08
	¼ MIC	22 ± 5.3	0.96	¼ MIC	264.7 ± 15	0.94
AcC14	½ MIC	25.7 ± 4	1.01	½ MIC	189 ± 1.4	0.95
	¼ MIC	24.5 ± 3.5	0.96	¼ MIC	194 ± 1	0.97
AcC16	½ MIC	28.3 ± 6.4	1.11	½ MIC	132.5 ± 13.4	0.66
	¼ MIC	23.7 ± 4.7	0.93	¼ MIC	107.5 ± 2.1	0.54
BrC12	½ MIC	50.5 ± 6.4	**1.98**	½ MIC	146 ± 18.5	1.15
	¼ MIC	20 ± 1.4	0.78	¼ MIC	179.3 ± 13.9	1.41
BrC14	½ MIC	21.5 ± 4,9	0.84	½ MIC	147.5 ± 21.9	1.16
	¼ MIC	18.7 ± 6.4	0.73	¼ MIC	158 ± 19.8	1.24
BrC16	½ MIC	47 ± 14.1	**1.84**	½ MIC	134 ± 1	1.06
	¼ MIC	70.5 ± 12	**2.76**	¼ MIC	151.3 ± 3.5	0.19
Positive control		65 ± 1	**2.55**		3200 ± 10	**7.1**
Negative control		22.8 ± 0.4			281 ± 14.01	

Significant values are in [bold].

### Hemolytic properties

**Figure 14:**
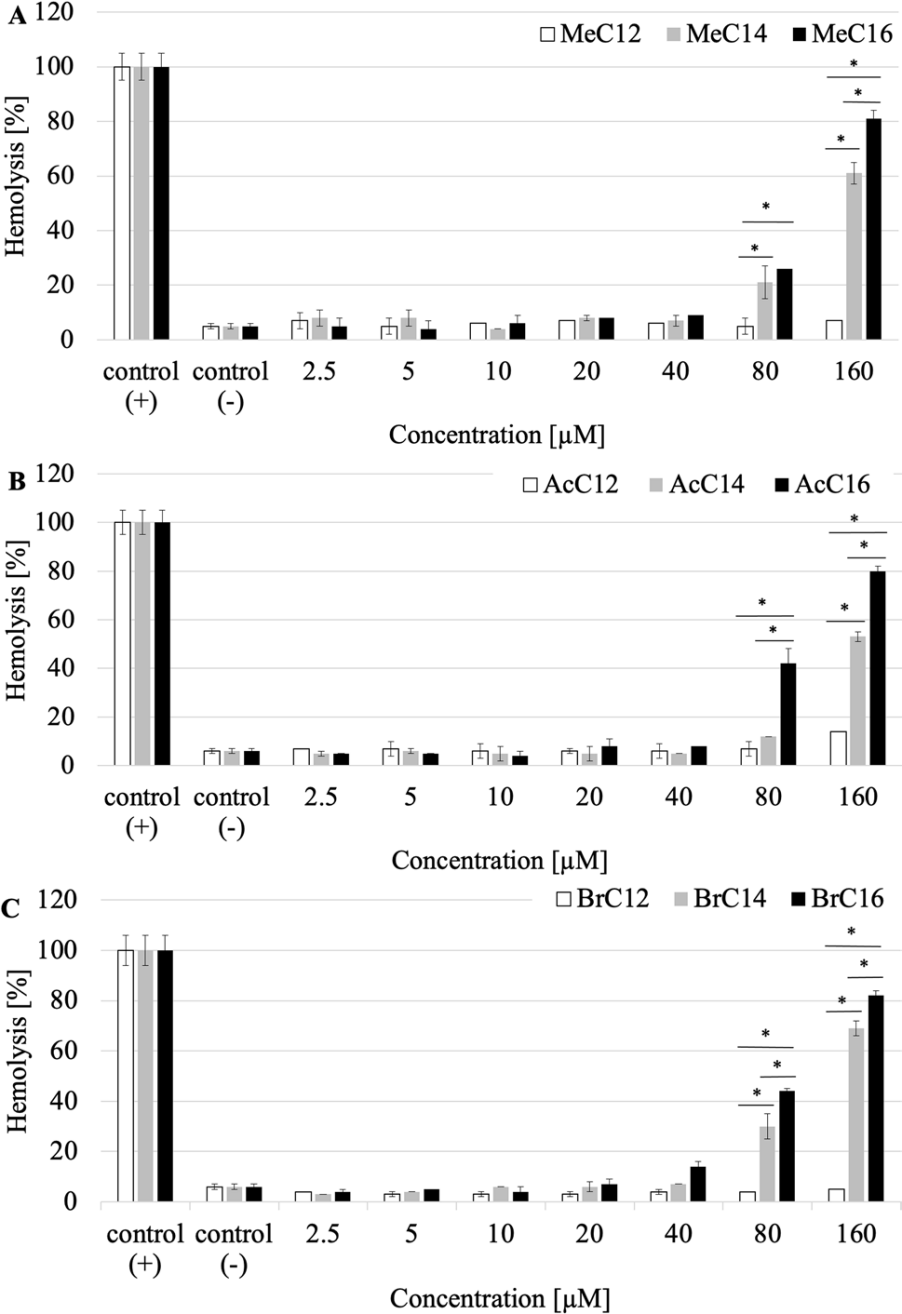
Hemolytic properties of QAS with counterion ((**A**) methylcarbonate Me, (**B**) acetate Ac, (**C**) bromide Br) and different chain lengths (C12, C14, C16); *significant difference between groups (p < 0.05).

Another necessary study for the applied use of the compounds is the analysis of the hemolytic properties of the tested QAS. Determination of hemolytic properties against animal erythrocytes is one of the most common tests in preliminary studies of chemicals with blood components^29^. Compounds with counterions such as methylcarbonate (Me), acetate (Ac) and bromide (Br) and chain lengths of C12, C14, C16 were tested. The analyses showed that the tested surfactants induced hemolysis of sheep cells only at high concentrations (80–1280 µM). The concentrations at which hemolysis was observed often exceeded the MIC concentrations of the compounds. It was observed that the longer the aliphatic chain of the compound was, the greater was the hemolysis of blood cells, regardless of the type of the counterion (Fig. 14).

## Discussion

Monomeric QAS are a class of surfactants composed of a single head and a single aliphatic chain. In their structure they contain a counterion which often increases the effect of the compound^8^. They show biological activity against pathogenic microorganisms not only in planktonic forms, but also against biofilms^30^. Due to the excessive use of surfactants, there is a problem of increasing resistance to disinfectants among microorganisms, including quaternary ammonium salts^31^.

The current work is an approach to select the most effective QAS in terms of antimicrobial activity against planktonic forms and biofilms. In addition, compounds inhibiting adhesion to the surface of stainless steel, polystyrene, glass, and silicone are also sought. The surfactants used in this work differed in aliphatic chain length (C12, C14, C16) and type of counterion (methylcarbonate, acetate, bromide). This selection allowed us to separate compounds which will be subjected to further stages of research, i.e., molecular analysis of mechanisms of action against *Saccharomyces cerevisiae* cells. In general, studies carried out on surfactants suggest that their main mechanisms involve perforation of cell membranes and cell disruption. However, it is reported^9,32^ that some QAS do not cause perforation of cell membranes but interact with them by changing their permeability. These interactions can lead to disruption of plasma membrane structure, resulting in leakage of intracellular material or alteration of plasma membrane function (e.g., proton gradient or secondary transport). Moreover, they can cause oxidative stress or affect lipid metabolism, resulting in accumulation of lipid droplets in the cytosol. Thus, the mechanisms of action may vary depending on the structure of the compound as well as on the structure of the cell envelopes of microorganisms.

One of the structural elements that has a great influence on the activity of the compounds is the length of the aliphatic chain. Mono-QAS with short chains (C2, C3) do not show biological activity. On the other hand, the optimal length of the hydrophobic carbon chain of compounds is determined from 12 to 18 carbon atoms^10^. The long alkyl chain can be built into the plasma membrane of microorganisms, while short chain surfactants may not have this ability. The highest effectiveness of QAS with long alkyl chain was observed against Gram-positive bacteria. Among Gram-negative bacteria, low activity of the compounds was observed, which is consistent with literature reports where bacterial envelopes are more difficult for the compounds to penetrate^10^. Reduced susceptibility among Gram-negative bacteria is determined not only by the presence of an outer membrane and lipopolysaccharide, but also by multiple resistance mechanisms, such as efflux pumps^33^. On the other hand, some studies suggest the compounds have activity against Gram-negative bacteria, fungi, and parasites^34,35^. Our study indicates that the antimicrobial activity of mono-QAS increases with aliphatic chain length. This observation is consistent with previous literature^35,36^. As reported by Gozzelino et al*.*^30^, mono-QAS with 16 carbon atoms in the chain showed greater activity against planktonic forms of *Escherichia coli*, *Staphylococcus aureus* and *Listeria monocytogenes* relative to compounds with shorter chains. Interestingly, this phenomenon is seen not only among mono-QAS but also among multifunctional compounds. Paluch et al.^32^ reported that multifunctional QAS with longer alkyl chains (C14, C16) exhibited greater antimicrobial potential. It is intriguing to note that among other gemini compounds, often higher activity was observed with shorter chain length^11,22^. These QAS have an additional head, an aliphatic chain, and a linker, which affect their ability to penetrate bacterial cell envelopes, which impact the antimicrobial activity of surfactants.

The strong cohesion between hydrophobic chains may be connected with presence of the secondary amide linker moiety with three methylene groups connecting the headgroup with the spacer, virtually lengthening the surfactant tail group. Presence of an N–H group in the secondary amide linker may also lead to occurrence of hydrogen bonding, resulting in strong cohesive forces between chains. Generally, moderation of strong hydrogen bonding, resulting in inferior water solubility and high values of Krafft points, i.e. the minimal temperature at which the surfactant exhibits sufficient activity in aqueous systems, may be performed by replacement of the secondary (i.e. containing an N–H bond) by a tertiary amide (e.g. containing an N–Me motif) moiety. The mentioned effect was widely studied for amphoteric 2-hydroxypropanesulfobetaine-type alkylamidopropyl derivatives^28^.

The most well-known, and probably the most commercially viable, example of cleavable surfactants comprises the family of cationic esterquat-type surfactants (often abbreviated as esterquats) with the ester bond –CO–O– or –O–CO– located between the quaternary ammonium head group(s) and the hydrocarbon tail(s)^37–39^. Grafting of the hydrolysable ester moiety onto the hydrophobic backbone of the surfactant structure allows decomposition of the molecule into fragments, lowering the environmental exposure levels and, furthermore, making it possible to improve the rate of biodegradation and to obtain high quality, environmentally friendly products for various applications. Moreover, using hydrolysable surfactants for formation of drug nanocarriers opens the possibility of designing new controlled delivery systems that can be activated by an internal or external triggering mechanism, as they are stable in aqueous solutions for a certain period only within a certain pH range. Amide bonds, like ester bonds, can be cleaved either by chemical hydrolysis or by enzyme catalyzed hydrolysis^15,37^. The amide bond is more stable than the ester bond to alkaline hydrolysis but is usually more susceptible to acid catalyzed cleavage. Amidases and peptidases are examples of amide-splitting enzymes. Lipases, which are normally associated with ester bond cleavage, sometime work also on amide bonds^40^. The chemical stability of the amide bond was found to be high. When the surfactant was subjected to 1 M sodium hydroxide for five days at room temperature, only 5% was cleaved. The corresponding experiment performed in 1 M HCl resulted in no hydrolysis. The amide bond was, however, found to be slowly hydrolyzed when lipase from *Candida antarctica* or peptidase was used as a catalyst. Amidase and lipase from *Mucor miehei* were found to be ineffective. Despite the very high chemical stability, the amide surfactant biodegraded by a similar path in the plot of biodegradation versus time as the corresponding ester surfactant, reaching 60% biodegradation within 28 days. Hence, it can be classified as readily biodegradable^40^. The mentioned facts clearly show that an amide linker in surfactants constitutes a good compromise between usefulness (i.e. chemical stability in basic, neutral and acidic conditions during storage and usage) and appropriate susceptibility to undergo biodegradation after use or in case of release to the environment.

Monomeric QAS are generally considered to be less active compared to multifunctional or gemini forms^10,11,41^. In the current study, compounds with 16 carbon atoms show efficacy against Gram-positive bacteria at low concentrations (MIC = 10 µM). The efficacy of 12- and 14-carbon compounds was lower. Compounds with longer aliphatic chains (C14, C16) also eradicated biofilms more effectively. Such a trend was also seen among multifunctional compounds, where compounds with 16 carbon atoms in the aliphatic chain better eradicated *C. albicans* biofilm^32^*.* This might be due to the fact that they penetrated and destabilized the biofilm matrix more effectively than compounds with shorter chains (C12).

QAS in low concentrations, below the CMC value, can form tiny aggregates called premicelles, which may decrease the activity of the compounds by reduction of their mobility.

Moreover, culture medium containing amphoteric components may influence this process.

The activity of quaternary ammonium salts is enhanced by counterions, in particular reactive ions, i.e. those having the ability to oxidize proteins or lipids. Among the most active are compounds possessing chlorine, bromide, or iron atoms^33,36,42^. In contrast, studies indicate that the counterions of mono-QAS compounds are not as important as the aliphatic chain length for the activity of the compound^36^. In our analyses, there was no clear effect of the nature of the counterion on antimicrobial activity against planktonic forms. However, mono-QAS having acetate or bromide in their structure were slightly more effective in eradicating the biofilm of all tested strains. In addition, bromide in the counterion most likely enhanced the mutagenic properties of compounds with 12 and 16 carbon atoms in the chain. Preventing the adhesion of microorganisms to surfaces is one method to prevent biofilm formation on abiotic surfaces. QAS have the ability to coat surfaces such as stainless steel, polystyrene, glass or silicone. These materials are used for catheters, endoprostheses, stents, and pacemakers, among other applications^19^. Therefore, it is extremely important to maintain their sterility throughout their lifetime^43,44^. One potential candidate is QAS or polymeric coatings containing immobilized QAS molecules^21^. Such a solution was applied by Kok et al.^45^ Application of quaternary ammonium silane significantly reduced *Enterococcus faecalis* and *Candida albicans* biofilm^45^. Studies have shown that compounds with longer chains (> C8) were generally more effective in preventing microbial adhesion. In the present study, methylcarbonates, acetates, and bromides with 16-carbon aliphatic chains (MeC16, AcC16 and BrC16) showed the highest efficacy among the nine compounds tested. This is most likely because most surfaces are naturally hydrophobic, so they interact with the hydrophobic aliphatic chain. The hydrophobicity of the compound will increase with the length of its chain^36,46^. As Paluch et al. point out^32^ the hydrophilic head of the compound via electrostatic interactions can repel the hydrophobic surface of the microorganism cell. Another possibility is the interaction of the cationic head of the compound with a negatively charged plasma membrane, leading to a change in the cell surface potential, thereby causing a disruption in its functioning^32^. Also, the research by Gozzelino et al*.*^30^ showed that mono-QAS with 16 carbon atoms in the chain effectively inhibited the adhesion of microorganisms to stainless steel. A compound with 8 carbon atoms in the chain also showed high effectiveness in their study^30^. Interactions that occur at the surface-QAS interface are the focus of surfactant research, as understanding the mechanisms of action is key to their application. It should be mentioned that some QAS can stimulate adhesion of microorganisms to surfaces, as has been reported by Paluch et al., Machado et al., and Ortiz et al.^32,47,48^.

For the future application of the compounds as medical plastic coatings, several studies are required that, among other things, would rule out their toxicity to higher organisms. Such studies include mutagenicity analysis and assessment of hemolytic properties of the compounds. Mono-QAS are considered to be non-toxic, biodegradable and environmentally safe compounds. Hence, in the form of ionic liquids they are called "green solvents". They are less toxic than dimeric, trimeric, or polymeric forms^10^. However, due to their nature, surfactants can penetrate the cell, thereby changing the osmolarity and causing cell lysis. Numerous studies indicate that the length of the aliphatic chain affects not only the antimicrobial activity of compounds, but also their toxicity^10,49–51^. In our study, hemolysis performed on animal blood cells is seen only at high compound concentrations exceeding the MIC. A similar result regarding other QAS was obtained by Xie et al*.*^52^, who noted a relationship between hemolytic potential and surfactant concentration^52^. According to the literature, it has also been observed that the longer the aliphatic chain is, the greater are the hemolytic properties. Therefore, it is necessary to invent compounds with optimal chain length in terms of toxicity to cells of higher organisms as well as antimicrobial activity. For example, Florio et al.^51,53^ observed that 12-carbon compounds with bromide in their structure (N-dodecyl-N-methylpyrrolidinium bromide, N-dodecyl-N-methylpiperidinium bromide) are characterized by high activity and low toxicity, which allowed application of the compounds in medicine [36][38]. Novel custom designed QAS type surfactants were designed and synthesized, providing such optimal structural parameters as the following: labile tertiary amide linker, able to moderate the hydrogen bonding within the surfactant molecule and to increase the aqueous solubility, varying hydrophobic tail and different counterions, including soft and reactive ones, good chemical stability, and potential biodegradability. The preparation methodology fulfills at least some principles of “green chemistry”—for application as disinfectants.

## Materials and methods

### Materials

#### Chemicals

All the used reagents (reagent or analytical grade) were used as received and obtained from Sigma-Aldrich, except *N*,*N*,*N*′-trimethyl-1,3-propanediamine (Alfa Chemistry). Deuterated solvents were purchased from Sigma-Aldrich. Organic solvents (analytical grade) were obtained from Avantor Performance Materials. Triethylamine was dried with solid calcium hydride (1 g per 50 mL of solvent) followed by distillation over this drying agent. Water used in all experiments was double-distilled (synthetic procedures) or purified by a Millipore (Bedford, MA) miliQ purification system (conductometric measurements).

#### Strains

The strains used in the study *S. epidermidis* ATCC 35984, *S. aureus* ATCC 6538, *P. aeruginosa* PAO1, *P. aeruginosa* ATCC 27853 came from the collection of the Department of Physico-Chemistry of Microorganisms of the Faculty of Biological Sciences of Wrocław University. Clinical strains *S. epidermidis* B374, *S. epidermidis* SI8, *S. aureus* MRSA R98, *E. coli* H64 come from the collection of the Medical University of Wrocław.

### Methods

#### ^1^H NMR analysis

^1^H NMR spectra were recorded on a Bruker AMX-500 spectrometer, using CDCl3 (chloroform-d) as a solvent (Tables 3, 4, 5). ^1^H chemical shifts (in ppm) were calibrated to TMS as an internal reference.

**Table 3:** ^1^H NMR analysis of linear alkylaminde-type quaternary ammonium bromides.

Abbreviation	Structure	^1^H NMR
BrC12		0.86–0.89 [t, 3H, –COCH_2_CH_2_(CH_2_)_8_**CH**_**3**_]; 1.22–1.34 [m, 16H, –COCH_2_CH_2_(**CH**_**2**_)_8_CH_3_]; 1.57–1.60 [m, 2H, –COCH_2_**CH**_**2**_(CH_2_)_8_CH_3_]; 2.09–2.13 [m, 2H, –N^+^CH_2_**CH**_**2**_CH_2_N–]; 2.31–2.34 [t, 2H, –CO**CH**_**2**_CH_2_(CH_2_)_8_CH_3_]; 3.09 [s, 3H, –N(**CH**_**3**_)–]; 3.43–3.55 [m, 11H; –**CH**_**2**_N^+^(**CH**_**3**_)_3_]; 3.64–3.67 [m, 2H; –N^+^CH_2_CH_2_**CH**_**2**_N–]
BrC14		0.85–0.87 [t, 3H, –COCH_2_CH_2_(CH_2_)_10_**CH**_**3**_]; 1.21–1.32 [m, 20H, –COCH_2_CH_2_(**CH**_**2**_)_10_CH_3_]; 1.58–1.61 [m, 2H, –COCH_2_**CH**_**2**_(CH_2_)_10_CH_3_]; 2.11–2.15 [m, 2H, –N^+^CH_2_**CH**_**2**_CH_2_N–]; 2.29–2.35 [t, 2H, –CO**CH**_**2**_CH_2_(CH_2_)_8_CH_3_]; 3.11 [s, 3H, –N(**CH**_**3**_)–]; 3.41–3.53 [m, 11H; –**CH**_**2**_N^+^(**CH**_**3**_)_3_]; 3.62–3.69 [m, 2H; –N^+^CH_2_CH_2_**CH**_**2**_N–]
BrC16r		0.84–0.88 [t, 3H, –COCH_2_CH_2_(CH_2_)_12_**CH**_**3**_]; 1.23–1.35 [m, 16H, –COCH_2_CH_2_(**CH**_**2**_)_12_CH_3_]; 1.59–1.62 [m, 2H, –COCH_2_**CH**_**2**_(CH_2_)_12_CH_3_]; 2.09–2.14 [m, 2H, –N^+^CH_2_**CH**_**2**_CH_2_N–]; 2.33–2.37 [t, 2H, –CO**CH**_**2**_CH_2_(CH_2_)_8_CH_3_]; 3.09 [s, 3H, –N(**CH**_**3**_)–]; 3.45–3.57 [m, 11H;—**CH**_**2**_N^+^(**CH**_**3**_)_3_]; 3.61–3.68 [m, 2H; –N^+^CH_2_CH_2_**CH**_**2**_N–]

**Table 4:** ^1^H NMR analysis of linear alkylaminde-type quaternary ammonium methylcarbonates.

Abbreviation	Structure	^1^H NMR
MeC12		0.86–0.89 [t, 3H, –COCH_2_CH_2_(CH_2_)_8_**CH**_**3**_]; 1.26–1.38 [m, 16H, –COCH_2_CH_2_(**CH**_**2**_)_8_CH_3_]; 1.57–1.60 [m, 2H, –COCH_2_**CH**_**2**_(CH_2_)_8_CH_3_]; 2.03–2.09 [m, 2H, –N^+^CH_2_**CH**_**2**_CH_2_N–]; 2.28–2.32 [t, 2H, –CO**CH**_**2**_CH_2_(CH_2_)_8_CH_3_]; 3.09 [s, 3H, –N(**CH**_**3**_)–]; 3.36 [s, 9H; –N^+^(**CH**_**3**_)_3_]; 3.44–3.57 [m, 7H; –N^+^**CH**_**2**_CH_2_**CH**_**2**_N–, C**H**_**3**_CO_3_^−^]
MeC14		0.86–0.89 [t, 3H, –COCH_2_CH_2_(CH_2_)_8_**CH**_**3**_]; 1.26–1.38 [m, 16H, –COCH_2_CH_2_(**CH**_**2**_)_8_CH_3_]; 1.57–1.60 [m, 2H, –COCH_2_**CH**_**2**_(CH_2_)_8_CH_3_]; 2.03–2.09 [m, 2H, –N^+^CH_2_**CH**_**2**_CH_2_N–]; 2.28–2.32 [t, 2H, –CO**CH**_**2**_CH_2_(CH_2_)_8_CH_3_]; 3.09 [s, 3H, –N(**CH**_**3**_)–]; 3.36 [s, 9H; –N^+^(**CH**_**3**_)_3_]; 3.44–3.57 [m, 7H; –N^+^**CH**_**2**_CH_2_**CH**_**2**_N–, C**H**_**3**_CO_3_^−^]
MeC16		0.86–0.89 [t, 3H, –COCH_2_CH_2_(CH_2_)_8_**CH**_**3**_]; 1.26–1.38 [m, 16H, –COCH_2_CH_2_(**CH**_**2**_)_8_CH_3_]; 1.57–1.60 [m, 2H, –COCH_2_**CH**_**2**_(CH_2_)_8_CH_3_]; 2.03–2.09 [m, 2H, –N^+^CH_2_**CH**_**2**_CH_2_N–]; 2.28–2.32 [t, 2H, –CO**CH**_**2**_CH_2_(CH_2_)_8_CH_3_]; 3.09 [s, 3H, –N(**CH**_**3**_)–]; 3.36 [s, 9H; –N^+^(**CH**_**3**_)_3_]; 3.44–3.57 [m, 7H; –N^+^**CH**_**2**_CH_2_**CH**_**2**_N–, C**H**_**3**_CO_3_^−^]

**Table 5:** ^1^H NMR analysis of linear alkylaminde-type quaternary ammonium acetates.

Abbreviation	Structure	^1^H NMR
AcC12		0.86–0.89 [t, 3H, –COCH_2_CH_2_(CH_2_)_8_**CH**_**3**_]; 1.26–1.38 [m, 16H, –COCH_2_CH_2_(**CH**_**2**_)_8_CH_3_]; 1.57–1.60 [m, 2H, –COCH_2_**CH**_**2**_(CH_2_)_8_CH_3_]; 2.03–2.22 [m, 2H, –N^+^CH_2_**CH**_**2**_CH_2_N–]; 2.28–2.32 [t, 2H, –CO**CH**_**2**_CH_2_(CH_2_)_8_CH_3_]; 3.07 [s, 3H, –N(**CH**_**3**_)–]; 3.37 [s, 9H; –N^+^(**CH**_**3**_)_3_]; 3.41–3.63 [m, 7H; –N^+^**CH**_**2**_CH_2_**CH**_**2**_N–, C**H**_**3**_COO–]
AcC14		0.86–0.89 [t, 3H, –COCH_2_CH_2_(CH_2_)_8_**CH**_**3**_]; 1.26–1.38 [m, 16H, –COCH_2_CH_2_(**CH**_**2**_)_8_CH_3_]; 1.57–1.61 [m, 2H, –COCH_2_**CH**_**2**_(CH_2_)_8_CH_3_]; 2.03–2.11 [m, 2H, –N^+^CH_2_**CH**_**2**_CH_2_N–]; 2.28–2.33 [t, 2H, –CO**CH**_**2**_CH_2_(CH_2_)_8_CH_3_]; 3.09 [s, 3H, –N(**CH**_**3**_)–]; 3.36 [s, 9H; –N^+^(**CH**_**3**_)_3_]; 3.44–3.64 [m, 7H; –N^+^**CH**_**2**_CH_2_**CH**_**2**_N–, C**H**_**3**_COO–]
AcC16		0.86–0.89 [t, 3H, –COCH_2_CH_2_(CH_2_)_8_**CH**_**3**_]; 1.26–1.38 [m, 16H, –COCH_2_CH_2_(**CH**_**2**_)_8_CH_3_]; 1.56–1.60 [m, 2H, –COCH_2_**CH**_**2**_(CH_2_)_8_CH_3_]; 2.03–2.09 [m, 2H, –N^+^CH_2_**CH**_**2**_CH_2_N–]; 2.25–2.36 [t, 2H, –CO**CH**_**2**_CH_2_(CH_2_)_8_CH_3_]; 3.12 [s, 3H, –N(**CH**_**3**_)–]; 3.38 [s, 9H; –N^+^(**CH**_**3**_)_3_]; 3.42–3.62 [m, 7H; –N^+^**CH**_**2**_CH_2_**CH**_**2**_N–, C**H**_**3**_COO^−^]

#### Synthesis of amideamine derivatives for linear-type quaternary ammonium bromides and methylcarbonates

*N*,*N*,*N*′-trimethyl-1,3-propanediamine (20.0 g, 0.1722 mol) was dissolved in 400 mL tetrahydrofuran:triethylamine (*v*:*v,* 1:1) mixture, after which the appropriate alkanoyl chloride (0.1722 mol: 37.67 g of dodecanoyl chloride, 42.59 g of tetradecanoyl chloride or 47.33 g of hexadecanoyl chloride) was added dropwise during intensive stirring. Then the reaction mixture was stirred for 6 h at room temperature, followed by filtration of triethylamine hydrochloride, the formed by-product. The filtrate then was evaporated and cautiously dried under reduced pressure for at least 4 h to get *N*-[3-(dimethylamine)propyl]-*N*-methyloalkylamides, namely *N*-[3-(dimethylamine)propyl]-*N*-methylododecanamide, *N*-[3-(dimethylamine)propyl]-*N*-methylotetradecanamide or *N*-[3-(dimethylamine)propyl]-*N*-methylohexadecanamide, as viscous liquids. Yield: 98–99.5%.

#### Synthesis of linear-type quaternary ammonium bromides

Appropriate *N*-[3-(dimethylamine)propyl]-*N*-methyloalkylamide (0.05 mol: 13.52 g of *N*-[3-(dimethylamine)propyl]-*N*-methylododecanamide, 16.33 g of *N*-[3-(dimethylamine)propyl]-*N*-methylotetradecanamide or 17.73 g of *N*-[3-(dimethylamine)propyl]-*N*-methylohexadecan- amide) was dissolved in 400 cm^3^ of dry tetrahydrofurane and kept at − 20 °C in tightly closed glass vessel for at least 3 h. Into the cooled solution bromomethane (0.1 mol, 9.5 g) was immediately added, followed by tightly closing of reaction vessel. The reaction was performed at 0–4 °C for 24 h without any stirring. The precipitates were filtered off, washed with dry tetrahydrofurane and dried over anhydrous P_2_O_5_ *in vacuo* for at least 12 h. The obtained [(3-dodecyilomethyloamine)propyl] trimethylammonium bromide, [(3-tetradecyilomethyloamine) propyl] trimethylammonium bromide and [(3-hexadecyilomethyloamine)propyl] trimethylammonium bromide were purified by recrystallization from methanol – ethyl acetate mixture and dried in desiccator over anhydrous P_2_O_5_. Yield: 80–90%.

#### Synthesis of linear-type quaternary ammonium methylcarbonates

Appropriate *N*-[3-(dimethylamine)propyl]-*N*-methyloalkylamide (0.122 mol: 36.42 g of *N*-[3-(dimethylamine)propyl]-*N*-methylododecanamide, 39.84 g of *N*-[3-(dimethylamine)propyl]-*N*-methylotetradecanamide or 43.26 g of *N*-[3-(dimethylamine)propyl]-*N*-methylohexadecanamide) and 0.610 mol (54,95 g) of dimethyl carbonate were placed in pressure reactor (Ecoclave, BuchiGlasUster, Switzerland) equipped with mechanical stirrer (Cyclone 300 ac, BuchiGlasUster, Switzerland), heating jacket and temperature probe (thermostating system Julabo BC4, JULABO GmbH, Germany). Reaction mixture was flushed with dry nitrogen four times, to remove any oxygen, and heated to 130 °C. Reaction was continued for 6 h at 130 °C and 750 rpm. After reaction completion the final mixture was collected and evaporated to dryness *in vacuo*. Pure [(3-dodecyilomethyloamine)propyl] trimethylammonium methylcarbonate, [(3-tetradecyilomethyloamine)propyl] trimethylammonium methylcarbonate and [(3-hexadecyilomethyloamine)propyl] trimethylammonium methylcarbonate were obtained by recrystallization from ethyl acetate. The desired products were filtered off, washed several times with ethyl acetate and dry, cold acetone and dried over anhydrous P_2_O_5_ *in vacuo* for at least 12 h. Yield: 65–75%.

#### Synthesis of linear-type quaternary ammonium acetates

[(3-dodecyilomethyloamine)propyl] trimethylammonium methylcarbonate (0.019 mol, 7.5 g), [(3-tetradecyilomethyloamine)propyl] trimethylammonium methylcarbonate (0.018 mol, 7.5 g) or [(3-hexadecyilomethyloamine)propyl] trimethylammonium methylcarbonate (0.017 mol, 7.5 g) was dissolved in 10 mL of anhydrous methanol and gentle warmed up to 30–40 °C under stripping with dry nitrogen. Into the obtained solution anhydrous lactic acid (around 1 mL) dissolved in 10 mL of anhydrous ethanol was dropwise added under continuous stirring and stripping with nitrogen. Reaction was continued for 4 h at 30–40 °C after addition of lactic acid solution completion. The final mixture was collected and evaporated to dryness *in vacuo*. Pure [(3-dodecyilomethyloamine)propyl] trimethylammonium acetate, [(3-tetradecyilomethylo- amine) propyl] trimethylammonium acetate or [(3-hexadecyilomethyloamine)propyl] trimethylammonium acetate were obtained by recrystallization from ethyl acetate. The desired product was filtered off, washed several times with diethyl ether and dried over anhydrous P_2_O_5_ *in vacuo* for at least 24 h. Yield: 65–90%.

#### Determination of critical micelle concentration by conductometric measurements

Values of critical micelle concentrations (CMC) of cationic surfactants were measured conductrometrically. N5721 conductometer (Tel-Eko Projekt sp z o.o., Poland), equipped with N5981 probe, was calibrated using 0.1 M, 0.01 M and 0.001 M KCl aqueous solutions (conductivities equal to 1.287 S/m, 0.141 S/m and 0.0147 S/m, respectively) and used without temperature compensation mode (constant temperature 25 °C was kept during all calibration procedures and measurements). Values of specific conductivities of at least 15 different concentrations for each surfactant were measured and plotted versus concentration; the discontinuity point was used to determine critical micelle concentration.

#### Minimal Inhibitory Concentration (MIC) and Minimal Bactericidal Concentration (MBC)

The minimum inhibitory concentration (MIC) of the tested monomeric QAS was performed using the microdilution method according to CLSI recommendation (M27-A3). Concentrations from 10 to 1280 µM were analyzed. Strains were incubated with or without compounds (growth control) for 24 h at 37 °C. Three independent replicates were performed. The MIC value was determined spectrophotometrically and the concentration that inhibited more than 90% of growth was determined. Optical density was measured at λ = 590 nm using a 96-well microplate reader (Bio-Rad Universal Hood II).

To determine the minimum bactericidal concentration (MBC), 10 µL of bacterial suspension incubated with monomeric QAS (MIC concentration and two times higher) was transferred to plates with LB (1% tryptone, 1% yeast extract, 0.5% NaCl) medium. Plates were incubated at 37 °C for 24 h and then colonies were counted. The concentration that reduced 99.9% of microbial growth was determined as MBC^54^.

#### Adhesion

##### Adhesion to polystyrene plate

Adhesion analysis to polystyrene surfaces was determined according to the method of Rodrigues et al. with modifications^55^. Polystyrene 96-well plates were incubated with the respective compound concentrations (10–1280 µM) for 4 h at 37 °C with 100 rpm shaking. The plates were then washed with miliQ and inoculated with 100 µL of bacterial suspension (OD = 0.4–0.6). Plates were incubated for 6 h at 37 °C with 100 rpm shaking. Wells were rinsed and fixed for 20 min at 60 °C. They were then decolorized with 0.1% crystal violet solution for 5 min. The wells were washed with miliQ until the unfixed dye was completely eluted. The absorbance at λ = 590 nm was measured using a reader (Bio-Rad Universal Hood II). The positive control was a surface not treated with QAS. Three independent trials were performed.

##### Adhesion to stainless steel, silicone, glass

Adhesion analysis to stainless steel was determined according to the method of Silva et al*.* with modifications^56^. For this purpose, stainless steel pads, catheters or cover glass were incubated together with appropriate concentrations of monomeric QAS (160–1280 µM) for 2 h at 37 °C with 100 rmp shaking. The surfaces were washed with miliQ and a bacterial suspension was added (OD = 0.4–0.6) and then incubated again for 4 h at 37 °C with 100 rmp shaking. The pads were washed with miliQ and transferred to phalcones with 5 mL PBS. Samples were sonicated (Bandelin SonoPuls HD 2070, 1 min × 3 cycles, 30%). Samples were diluted (10^–3^, 10^–4^) and suspended 100 µL per LB. They were incubated at 37 °C for 24 h. Colonies were then counted. The positive control was a surface not treated with QAS. Three independent trials were performed.

#### Biofilm eradication

Biofilm eradication assay was performed according to the method of Christensen et al*.* with modifications^57^. A standardized bacterial (OD = 0.6) suspension of 100 µL was spotted into a 96-well polystyrene plate. They were incubated for 24 h at 37 °C. Cultures were removed, the plate was washed and then concentrations of QAS (10–1280 µL) were added, incubated for 4 h at 37 °C with 100 rpm shaking. The wells were washed with miliQ and fixed at 60 °C for 20 min. The wells were decolorized with 0.1% crystal violet solution for 5 min. The wells were washed with miliQ until the unfixed dye was completely eluted. The absorbance at λ = 590 nm was measured using a reader (Bio-Rad Universal Hood II). The positive control was an untreated biofilm. Three independent test trials were performed.

#### Biofilm-oriented antiseptics test (BOAT)

Biofilm-oriented antiseptics test (BOAT) was performed according to the method of Junka et al*.*^58^. The strain (*S. epidermidis* B374) was cultured into an appropriate liquid TSB medium and incubated at 37 °C for 24 h. After incubation, the bacterial suspension was diluted with fresh medium to optical density OD = 0.125 (λ = 590). A dilution 10^–3^ was made a and transferred to 96-well polystyrene plate. Next, the suspensions were incubated at 37 °C for 24 h. After 24 h, the suspensions from both plates were removed and thoroughly rinsed with 0.9% NaCl. Next, 100 μL of undiluted (working solution) of QAS (AcC16) in a certain concentration was transferred to the well for selected contact time (30 min and 60 min). After the contact time, the antiseptic was removed and the wells were filled with an appropriate universal neutralizing agent (Saline Peptone Water, Biocorp, Warsaw, Poland) for 5 min. After this time, the neutralizing agent was removed. The wells were filled with 100 μL of an appropriate medium and with 5 μL of tetrazolium chloride (TTC) (Fluka, Poznan, Poland), a reagent staining metabolically active microorganisms red. The results were assessed colorimetrically after 24 h of incubation of the plate at 37 °C. Samples were diluted and suspended 100 µL per LB and incubated at 37 °C. Colonies were then counted. The positive control was an untreated biofilm, confirming the ability of the test strain to form biofilm. The negative control wells filled with medium (no biofilm) confirming the sterility of the test performer and TTC.

#### Mutagenicity

Two reference strains of *Salmonella* Typhimurium TA98 and *Salmonella* Typhimurium TA100, deficient in histidine synthesis, were used for this study according to the method proposed by Ames et al*.* with modifications^59^. 100 µL bacterial culture (OD = 1.5), 100 µL compound of a specific concentration (1/2 MIC, 1/4 MIC) and 200 µL biotin (0.031%) with histidine (0.024%) solution were added to 2 mL top agar. The mixture was mixed and poured onto a Davis minimal medium plate. The mixture without the test compound was used as a negative control. The positive control was a mixture of bacterial culture, biotin-histidine solution, and sodium azide (at a concentration of 15 μg/mL for strain TA100) or acriflavine (at a concentration of 100 μg/mL for strain TA98). Plates were incubated for 48 h at 37 °C, and then colonies were counted. The mutagenicity ratio (MR), the ratio of the number of revertants grown in the presence of the test compound to the number of spontaneously occurring revertants (negative control), was calculated. A mutagenic ratio equal to or greater than 1.7 indicates the mutagenic potential of the tested compounds. The study was performed in three times.

#### Hemolytic properties

QAS was assayed for hemolytic activity as described by Falkinham III et al.^60^*.* Sheep blood (5 mL) was centrifuged for morphotic elements (2500 rpm, 15 min), washed three times in PBS (pH 7.4) and resuspended in PBS. Compounds of different concentrations in a volume of 10 µL (2.5 – 1280 µL) were transferred to the wells of a titer plate and mixed with 90 µL of erythrocytes. They were incubated for 1.5 h at 37 °C. Centrifuged (2500 rpm, 15 min), and the supernatant was transferred to a sterile flat-bottomed titer plate. The absorbance at λ = 540 was measured (Bio-Rad Universal Hood II). PBS and 1% SDS were used as positive and negative controls, respectively. This assay was repeated three times.

#### Statistical analysis

The results of all the experiments are given as a mean value ± SD (standard deviation) of three independent experiments. The differences in adhesion to different surfaces, biofilm eradication, hemolysis properties of QAS were analyzed with a parametric t-test for independent samples using Statistica v. 13 software (StatSoft, Krakow, Poland). Differences between groups were considered statistically significant for p values < 0.05.

## Supplementary Information

Supplementary Information.

## Data Availability

The datasets used and/or analysed during the current study available from the corresponding author on reasonable request. All samples are available from the authors upon request.
