# Supplementary material for: The effectiveness of newly synthesized quaternary ammonium salts differing in chain length and type of counterion against priority human pathogens
Source: Sci Rep. 2022 Dec 16;12:21799. doi: 10.1038/s41598-022-24760-y (PMC9757636; doi:10.1038/s41598-022-24760-y)
Supplement: Supplementary file 1 — Supplementary Information. [file 41598_2022_24760_MOESM1_ESM.docx]

**ESI:**

**ELECTRONIC SUPPLEMENTARY MATERIAL**

**The effectiveness of newly synthesized quaternary ammonium salts (QASs) differing in chains length and the type of counterion against priority human pathogens**

*1. Adhesion of microorganisms to silicone and glass*


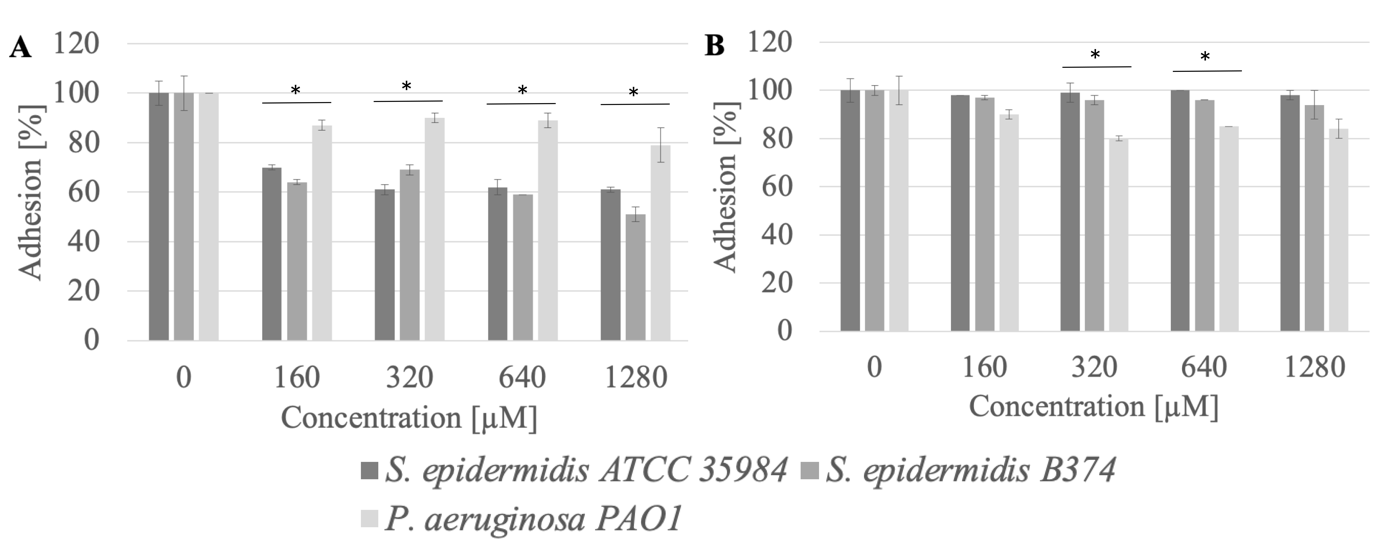


Figure S1. Adhesion of microorganisms to surfaces (A - silicone, B - glass) after incubation with MeC16 compound; * significant difference between groups (p<0.05).


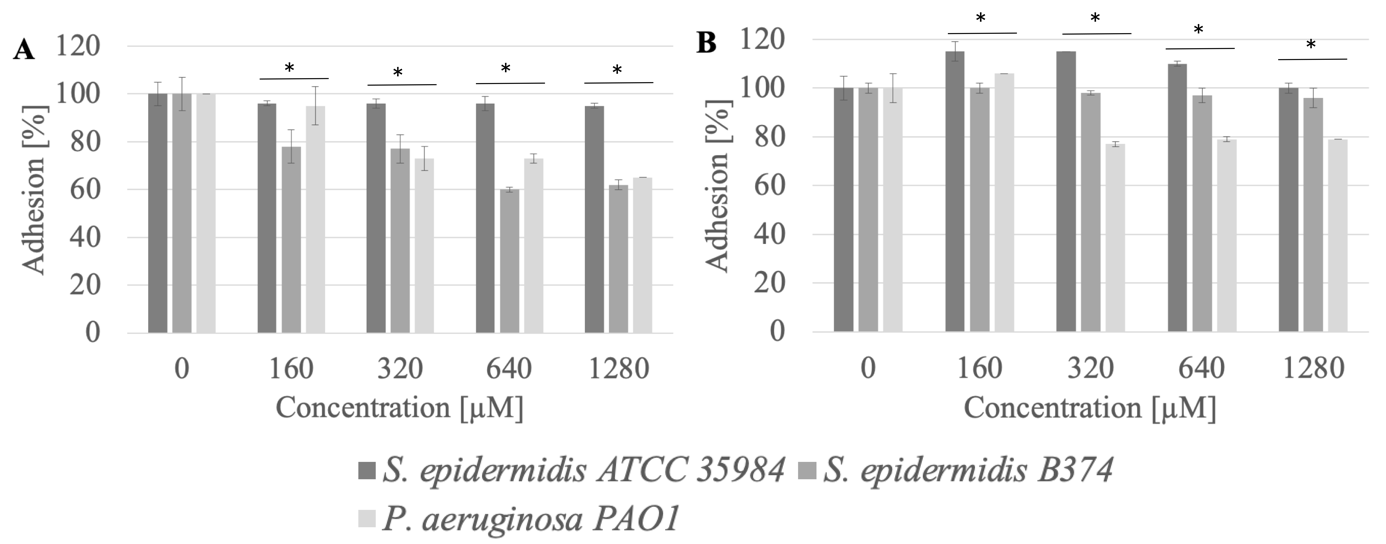


Figure S2. Adhesion of microorganisms to surfaces (A - silicone, B - glass) after incubation with MeC12 compound; * significant difference between groups (p<0.05).


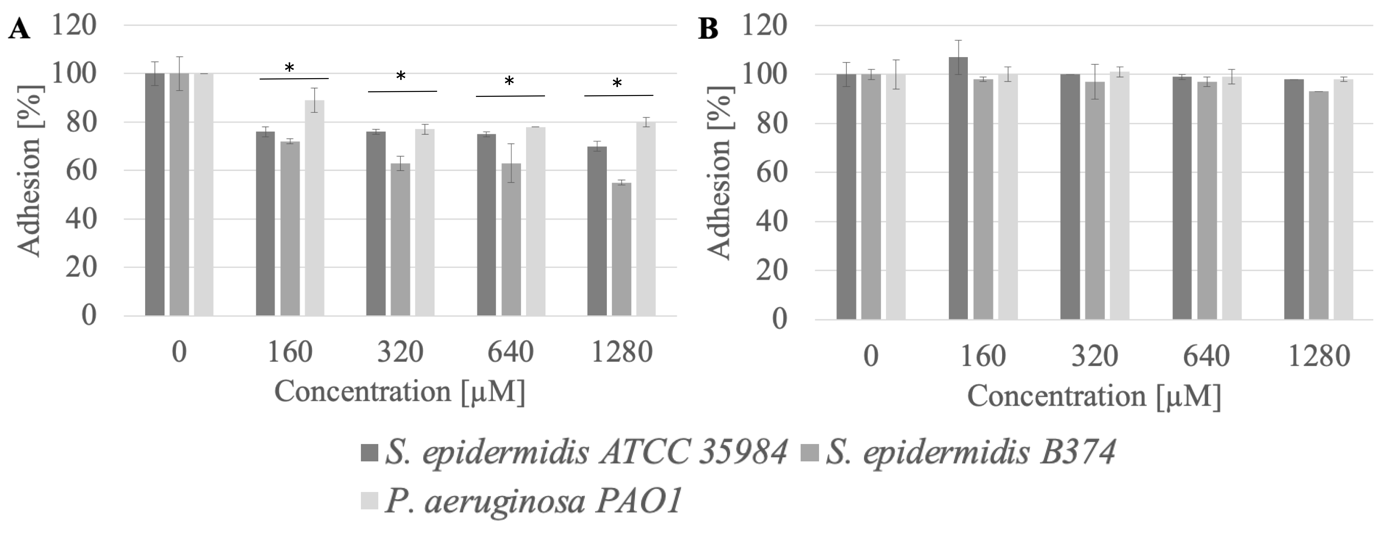


Figure S3. Adhesion of microorganisms to surfaces (A - silicone, B - glass) after incubation with MeC14 compound; * significant difference between groups (p<0.05).


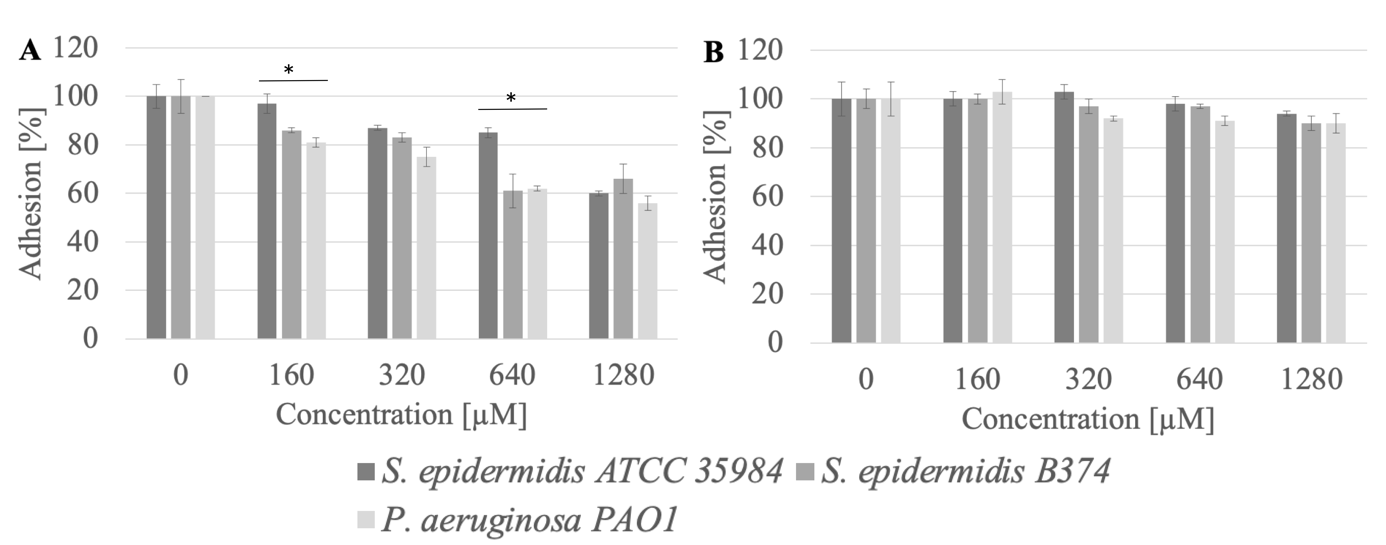


Figure S4. Adhesion of microorganisms to surfaces (A - silicone, B - glass) after incubation with AcC16 compound; * significant difference between groups (p<0.05).


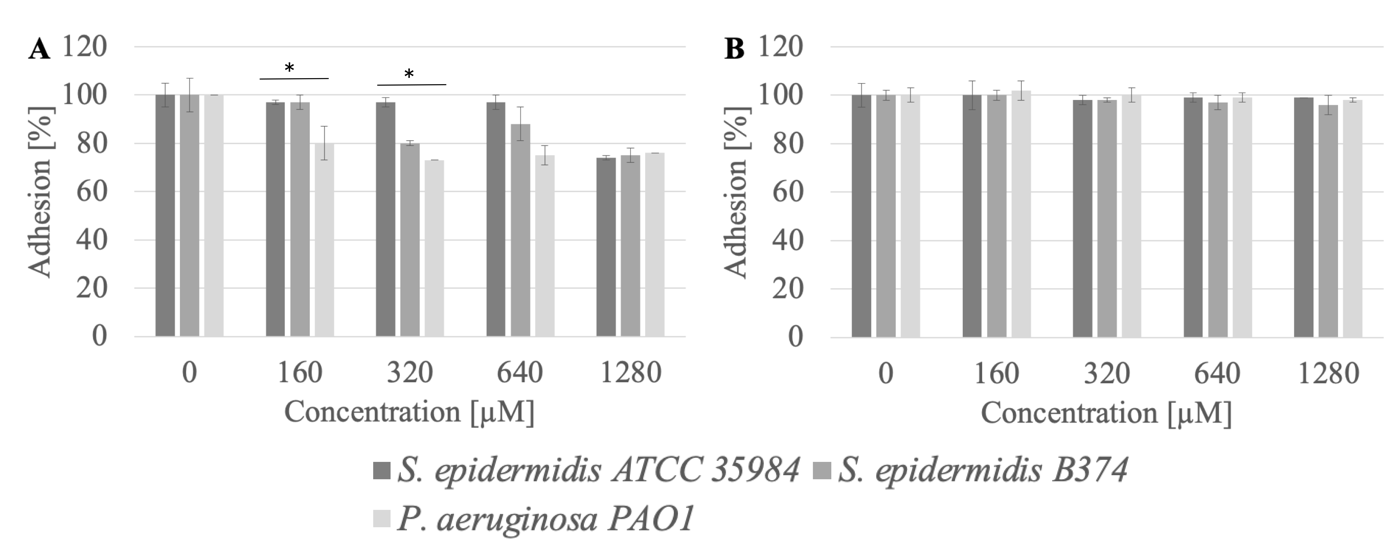


Figure S5. Adhesion of microorganisms to surfaces (A - silicone, B - glass) after incubation with AcC12 compound; * significant difference between groups (p<0.05).


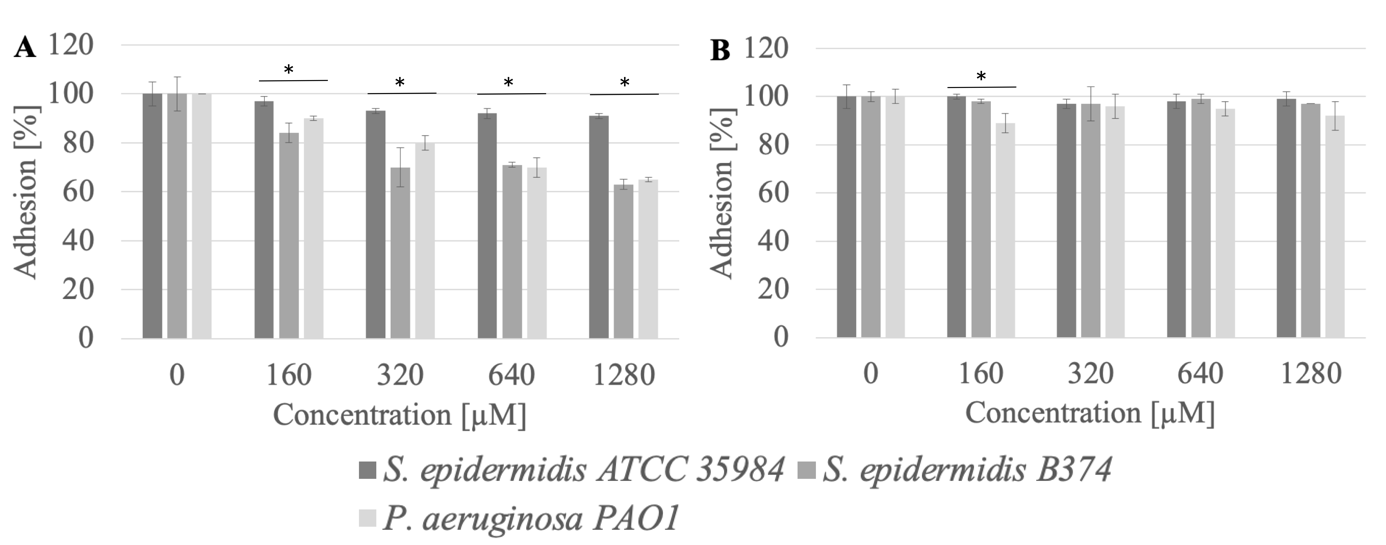


Figure S6. Adhesion of microorganisms to surfaces (A - silicone, B - glass) after incubation with AcC14 compound; * significant difference between groups (p<0.05).


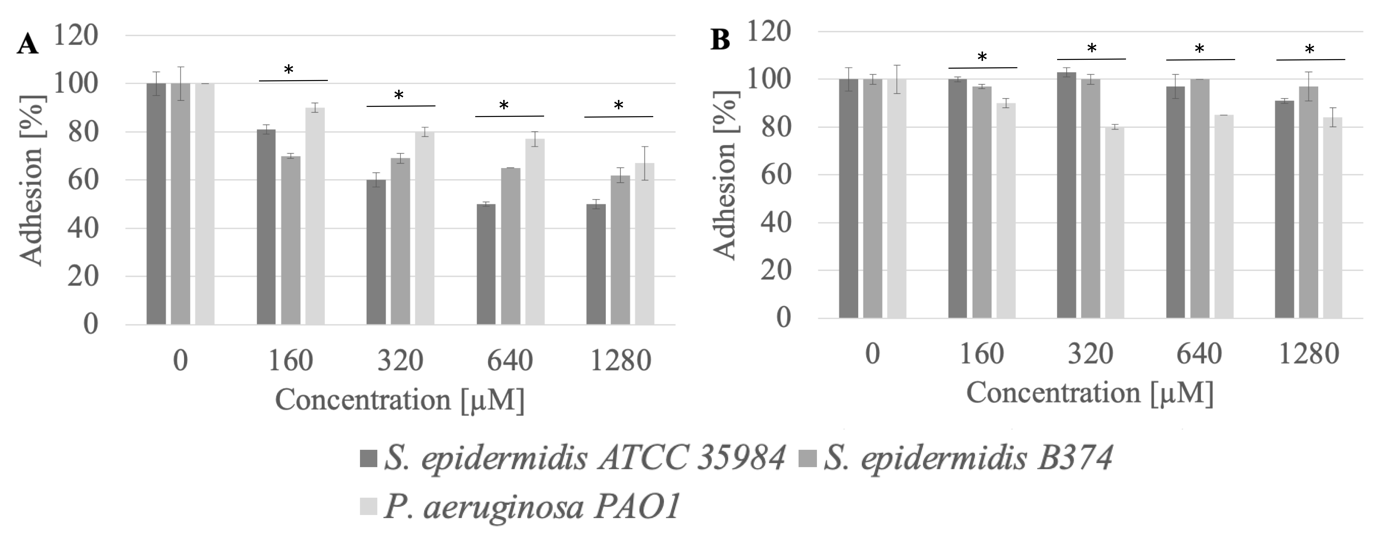


Figure S7. Adhesion of microorganisms to surfaces (A - silicone, B - glass) after incubation with BrC16 compound; * significant difference between groups (p<0.05).


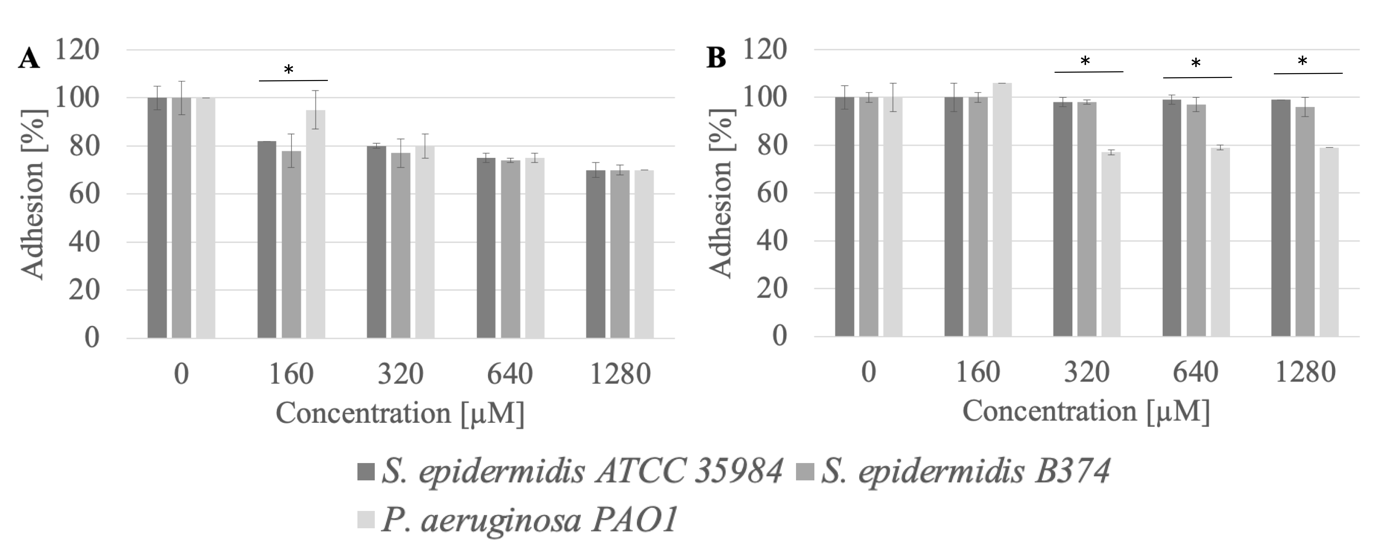


Figure S8. Adhesion of microorganisms to surfaces (A - silicone, B - glass) after incubation with BrC12 compound;* significant difference between groups (p<0.05).


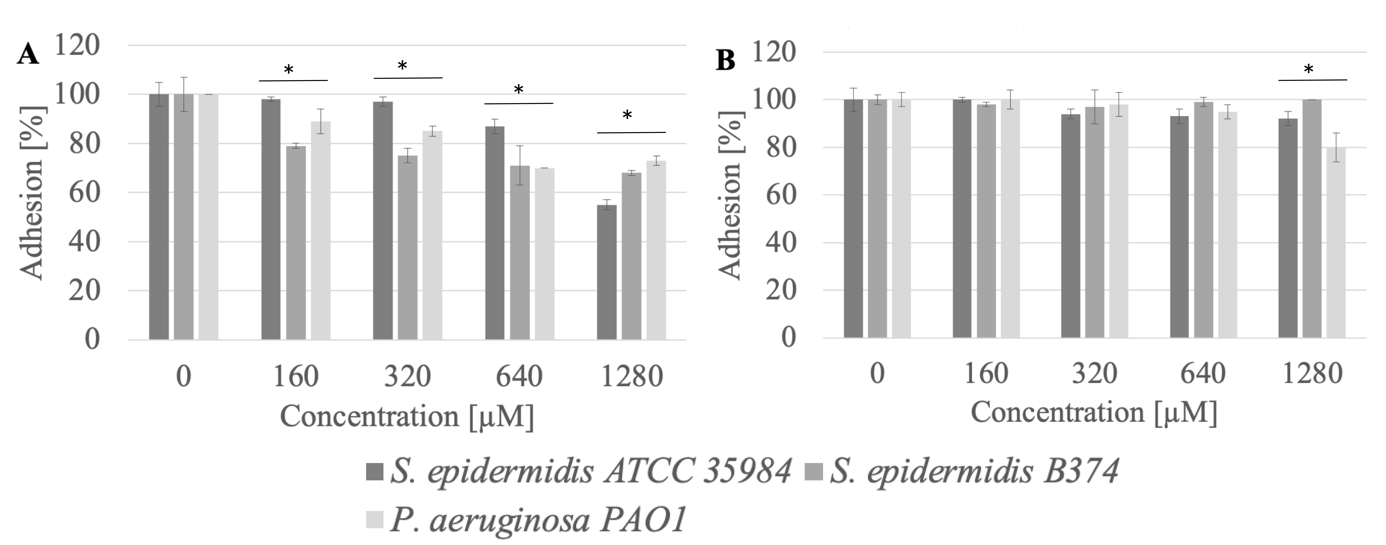


Figure S9. Adhesion of microorganisms to surfaces (A - silicone, B - glass) after incubation with BrC14 compound; * significant difference between groups (p<0.05).

*2. Determination of CMC values of the studied surfactants by the conductometric method.*


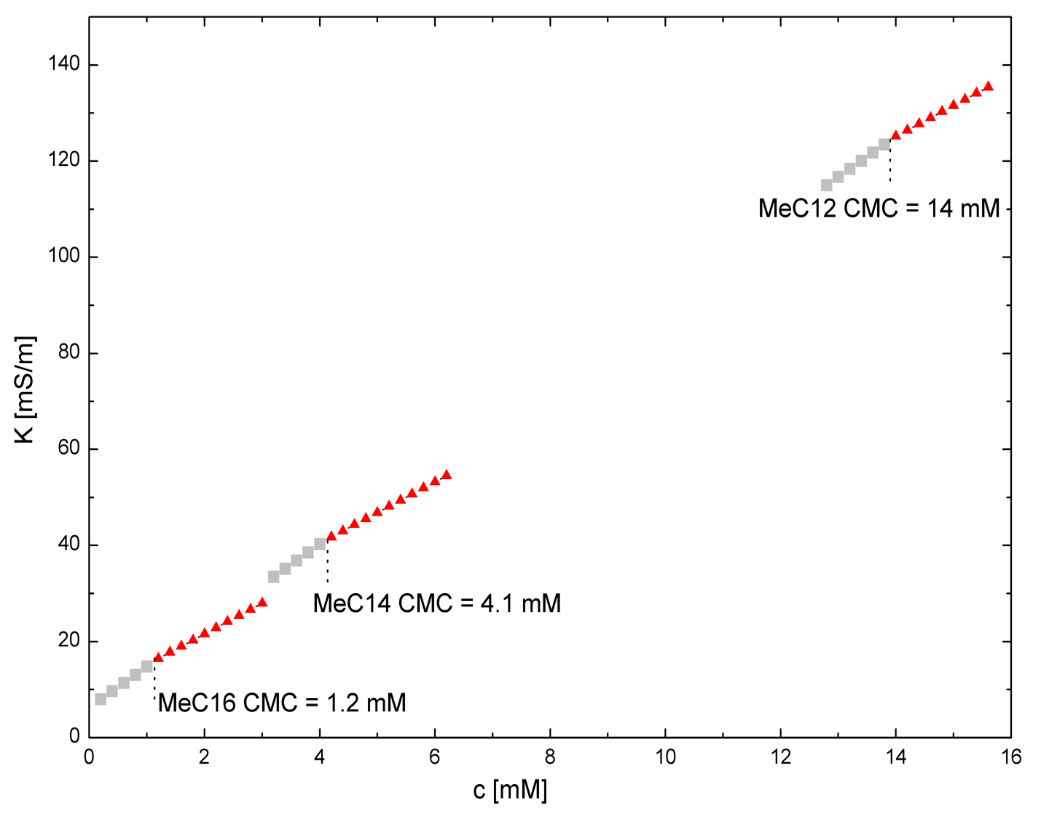


Figure S10. Electric conductivity vs. concentration of MeC12, MeC14 and MeC16 in aqueous solution at 25°C.


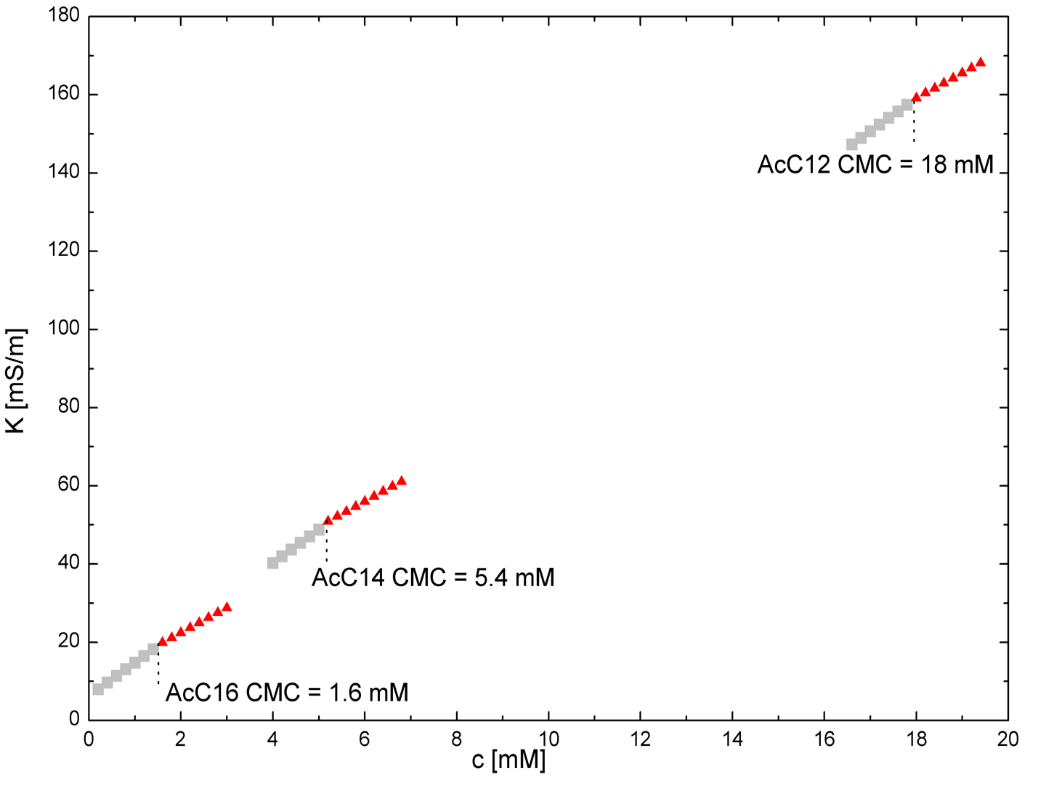


Figure S11. Electric conductivity vs. concentration of AcC12, AcC14 and AcC16 in aqueous solution at 25 °C.


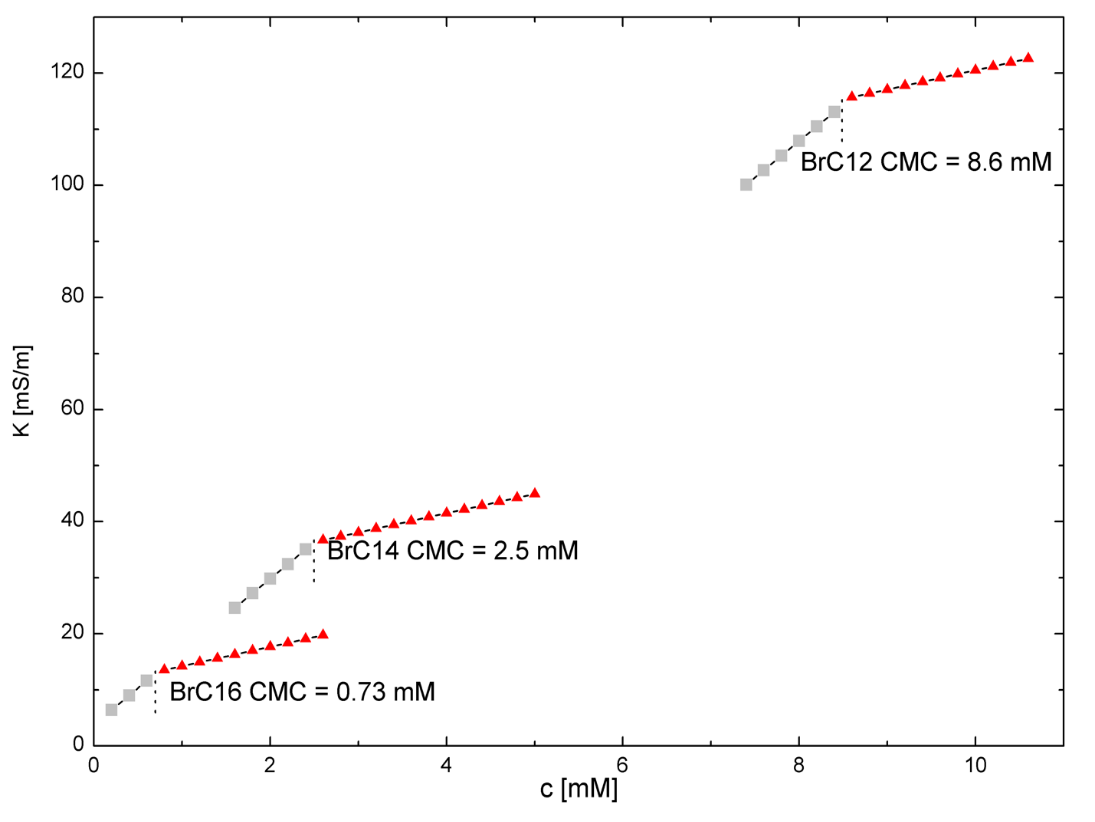


Figure S12. Electric conductivity vs. concentration of BrC12, BrC14 and BrC16 in aqueous solution at 25 °C.


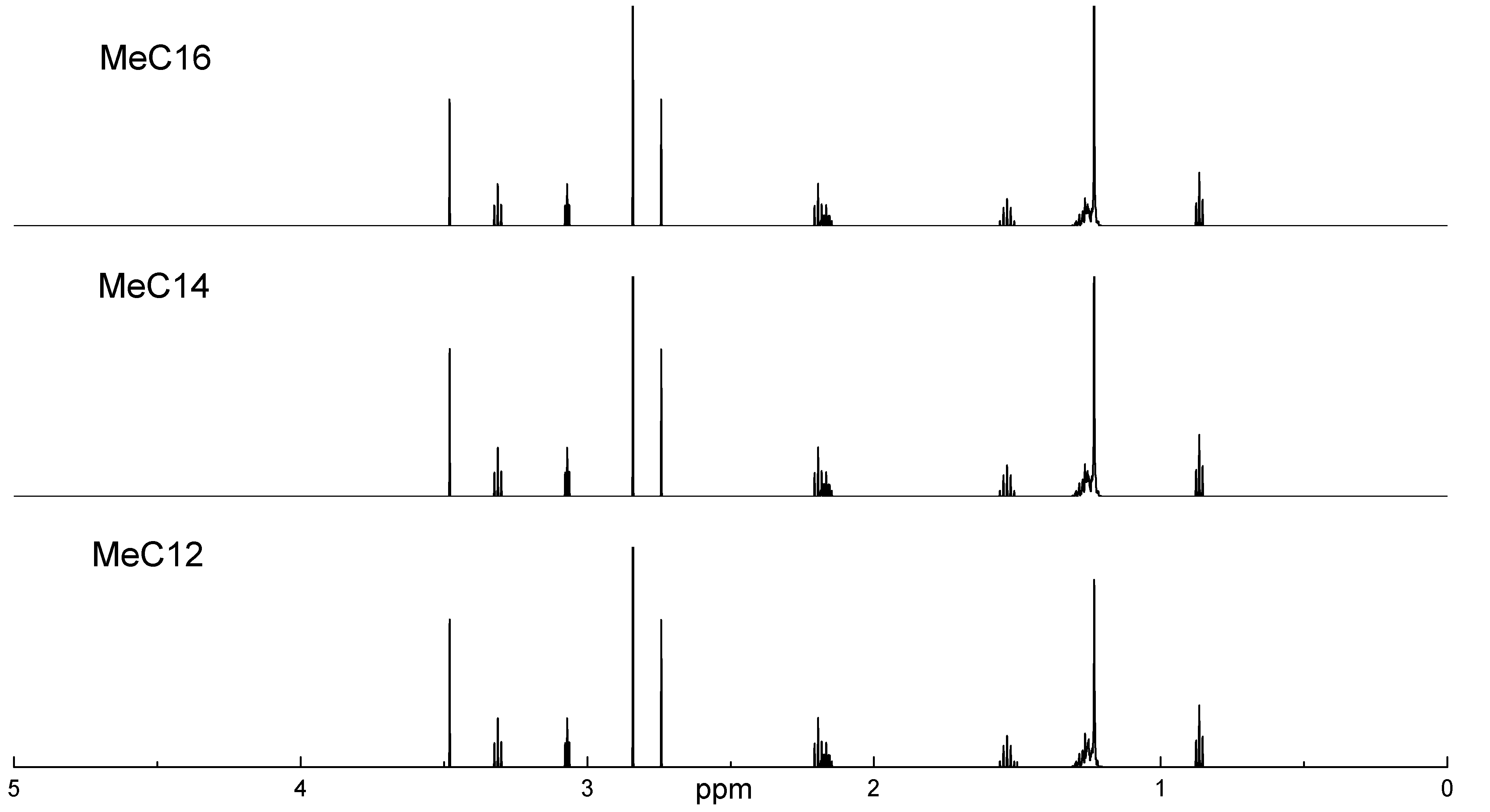


Figure S13. ^1^H MNR spectra of MeC12, MeC14 and MeC16.


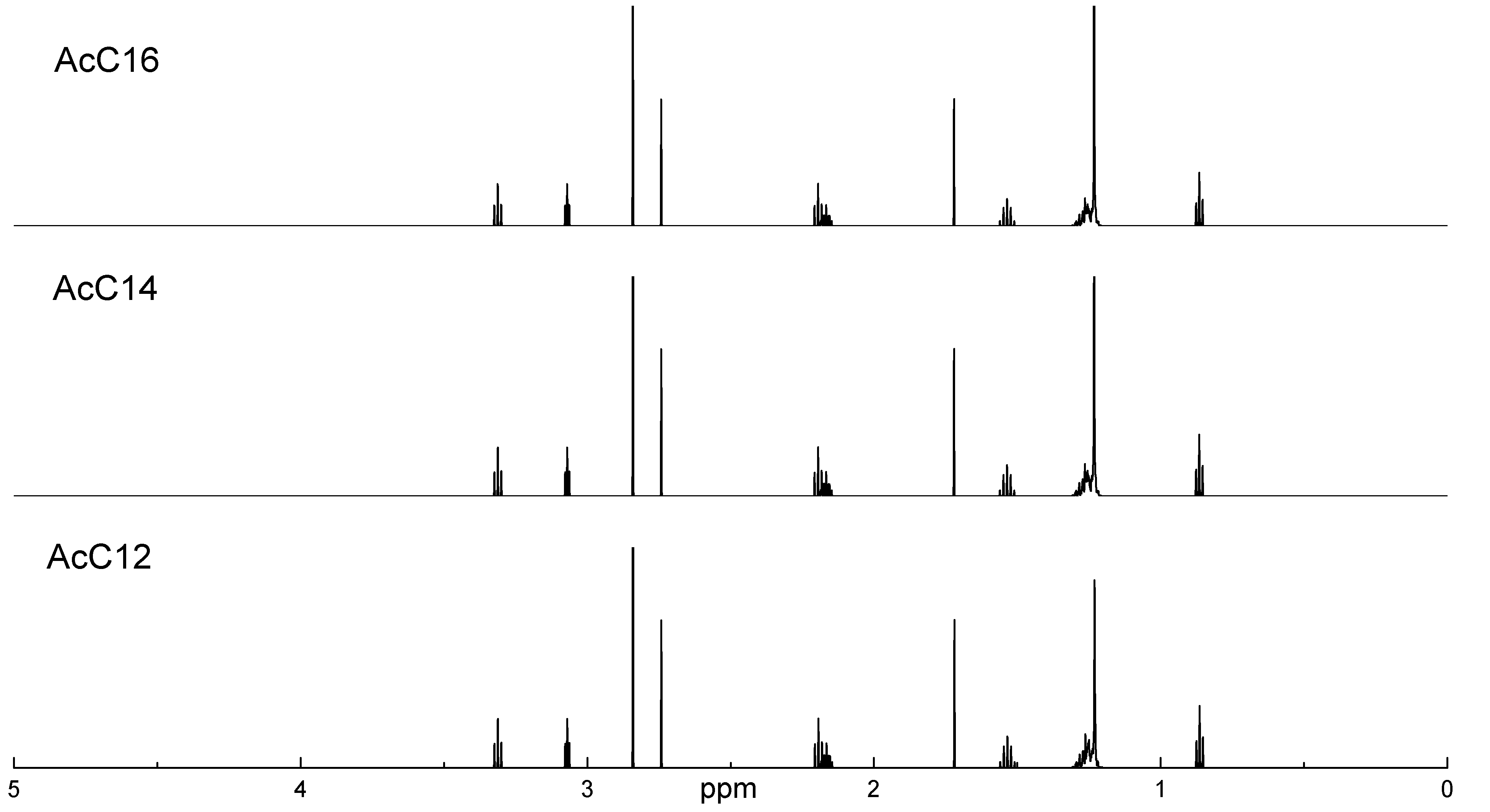


Figure S14. ^1^H MNR spectra of AcC12, AcC14 and AcC16.


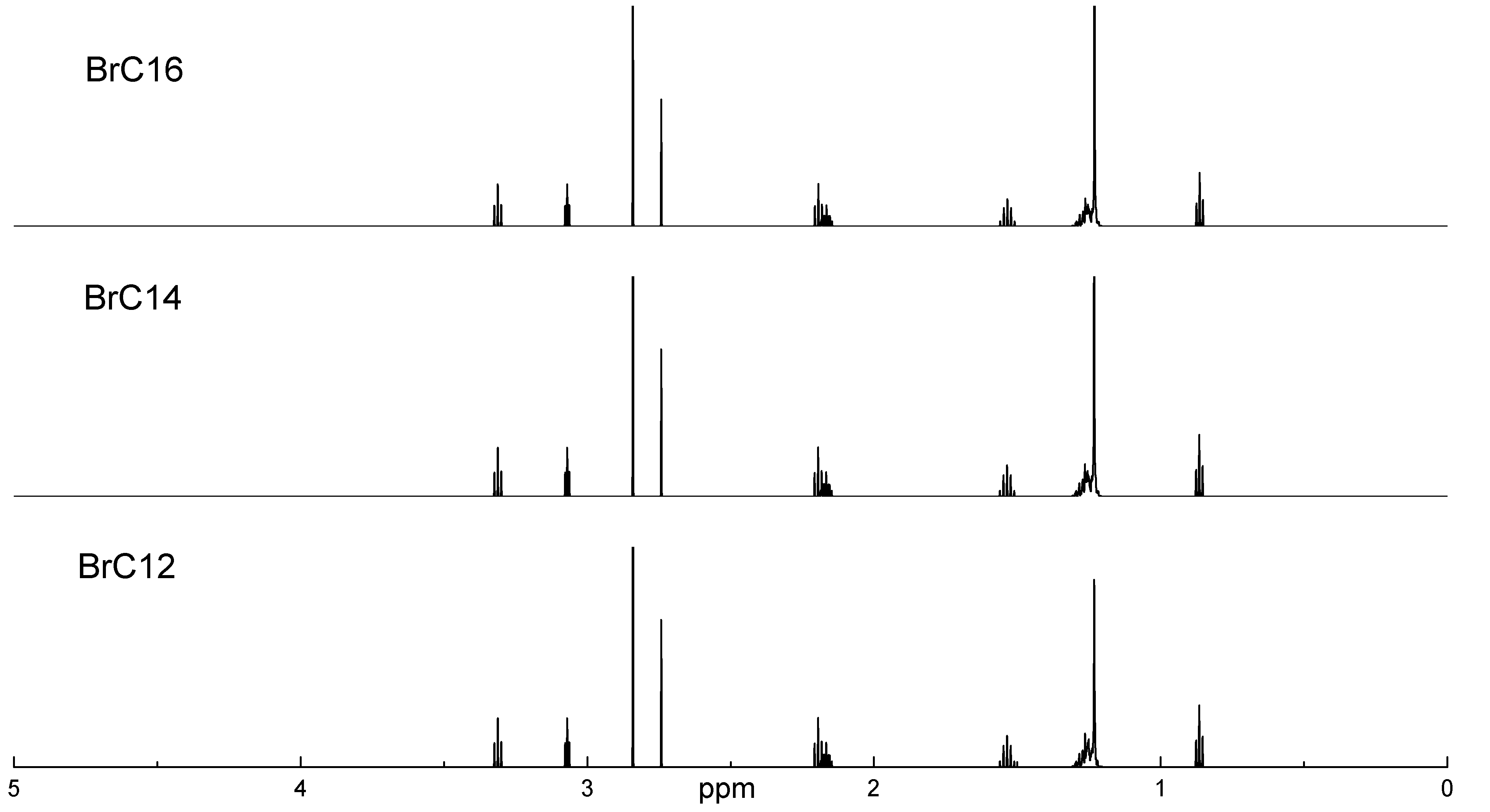


Figure S15. ^1^H MNR spectra of BrC12, BrC14 and BrC16.
